# Assessment of two brands of fentanyl test strips with 251 synthetic opioids reveals “blind spots” in detection capabilities

**DOI:** 10.1186/s12954-023-00911-w

**Published:** 2023-12-06

**Authors:** Kathleen L. Hayes, Marya Lieberman

**Affiliations:** Department of Chemistry and Biochemistry, University of Notre Dame, Notre Dame, IN 46556 USA

**Keywords:** Fentanyl, Fentanyl analogs, Drug checking, Harm reduction, Fentanyl test strips, Lateral flow immunoassays

## Abstract

**Background:**

Fentanyl test strips (FTS) are a commonly deployed tool in drug checking, used to test for the presence of fentanyl in street drug samples prior to consumption. Previous reports indicate that in addition to fentanyl, FTS can also detect fentanyl analogs like acetyl fentanyl and butyryl fentanyl, with conflicting reports on their ability to detect fentanyl analogs like Carfentanil and furanyl fentanyl. Yet with hundreds of known fentanyl analogs, there has been no large-scale study rationalizing FTS reactivity to different fentanyl analogs.

**Methods:**

In this study, 251 synthetic opioids—including 214 fentanyl analogs—were screened on two brands of fentanyl test strips to (1) assess the differences in the ability of two brands of fentanyl test strips to detect fentanyl-related compounds and (2) determine which moieties in fentanyl analog chemical structures are most crucial for FTS detection. Two FTS brands were assessed in this study: BTNX Rapid Response and WHPM DanceSafe.

**Results:**

Of 251 screened compounds assessed, 121 compounds were detectable at or below 20,000 ng/mL by both BTNX and DanceSafe FTS, 50 were not detectable by either brand, and 80 were detectable by one brand but not the other (*n* = 52 BTNX, *n* = 28 DanceSafe). A structural analysis of fentanyl analogs screened revealed that in general, bulky modifications to the phenethyl moiety inhibit detection by BTNX FTS while bulky modifications to the carbonyl moiety inhibit detection by DanceSafe FTS.

**Conclusions:**

The different “blind spots” are caused by different haptens used to elicit the antibodies for these different strips. By utilizing both brands of FTS in routine drug checking, users could increase the chances of detecting fentanyl analogs in the “blind spot” of one brand.

**Supplementary Information:**

The online version contains supplementary material available at 10.1186/s12954-023-00911-w.

## Background

Fentanyl is a potent; FDA-approved synthetic opioid used since 1972 for treating pain [1]. Since its discovery, illicit consumption of fentanyl has risen sharply and, along with its analogs, was responsible for majority of the 70,601 synthetic opioid-caused overdose deaths in the United States in 2021 [2]. Fentanyl analogs—or fentalogs—are derivatized fentanyls with modifications that can increase or decrease potency; some were developed for pharmaceutical and veterinary use (Carfentanil, sufentanil, alfentanil, remifentanil) [3, 4] while others were—and continue to be—produced illicitly for recreational drug use (α-methyl fentanyl, 3-methyl fentanyl, thiofentanyl, acetyl fentanyl, butyryl fentanyl) [5–8]. Fentanyl analogs pose a unique challenge to comprehensive drug screening, as new compounds are constantly emerging and standard targeted drug checking methods like GC–MS and LC–MS/MS will fail to detect them until their reference standards catch up. For this reason, it is believed that the severity of fentanyl analog outbreaks is underreported, including 2013 acetyl fentanyl outbreaks in Rhode Island, Pennsylvania, and North Carolina [4, 9, 10]. 

To reduce the harm associated with illicit drug use, federal funds have been allocated for fentanyl test strip distribution by the Centers for Disease Control and Prevention (CDC) and the Substance Abuse and Mental Health Services Administration (SAMHSA) [11, 12]. Fentanyl test strips (FTS) are lateral flow immunoassays that rapidly detect fentanyl in solution. At about $1 USD per test, FTS are an accessible alternative to other drug checking methods that require expensive instrumentation or trained personnel. Briefly, FTS function as a competitive immunoassay. As the sample flows across the device, it first encounters color-labeled competitive binding particles. As the sample flows across the test region, any target analyte present (i.e., fentanyl) will be captured by the immobilized monoclonal test antibody, preventing the color-labeled competitive binding particles from being captured. A control region containing immobilized control antibodies binds color-labeled particles [13]. Ultimately a single line in the control area indicates a positive result—fentanyl *is* present in the samples—while two lines, no matter how faint the test line, indicate a negative result- fentanyl is *not* detectable in the solution.

Though originally developed to detect fentanyl in urine, FTS have been utilized for off-label harm reduction purposes as a low-cost, easy-to-use drug checking tool. There have been multiple studies on the efficacy of BTNX Rapid Response FTS for testing street drug samples for fentanyl. These reports cite incidence of false positives between 0 and 9.6% and false negatives between 3.7 and 10.9%, and limits of detection between 100 and 200 ng/mL [14–18]. Cross-reactivity of FTS with methamphetamine, MDMA, and diphenhydramine—a common cutting agent in illicit drugs—is a major drawback of BTNX Rapid Response FTS but ultimately researchers have concluded that FTS are a valid and useful tool for drug checking [14, 19]. In part due to the relatively large amount of research on the product, BTNX Rapid Response FTS have been the major brand adopted by harm reduction organizations for drug checking. Yet as demand for FTS has grown, more products have entered the market including DanceSafe FTS, manufactured by WHPM, which claims to use a higher specificity antibody that does not produce false positives by MDMA, methamphetamine, or cocaine [20]. Currently, no federal agencies regulate FTS for drug checking in the US.

**Fig. 1:**
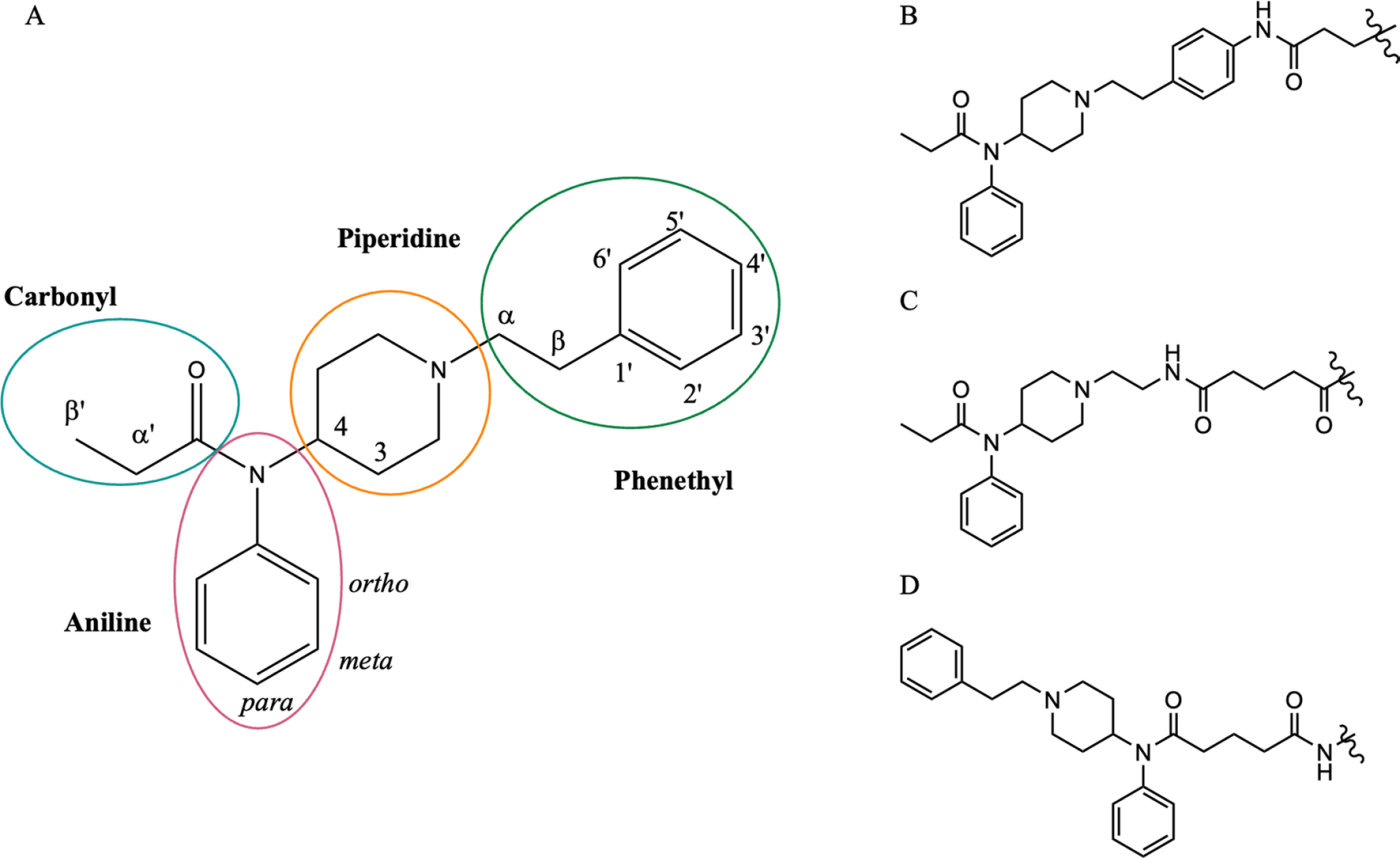
Fentanyl structure with labeled moieties and carbons (**A**) and fentanyl haptens reported by Barrientos [21, 22] (**B**), Raleigh [23] (**C**), and Haile [24] (**D**)

The identities of the monoclonal antibodies used on BTNX and DanceSafe FTS to target fentanyl are proprietary, as are the details of their creation. However, since fentanyl is too small to elicit an immune response on its own, use of fentanyl haptens is necessary for antibody development. These haptens structurally resemble fentanyl (Fig. 1A) and are conjugated to a large carrier molecule, like a protein, that can stimulate antibody production. Although the haptens used for BTNX and DanceSafe FTS development are unknown, there is literature precedent that different fentanyl haptens can lead to effective anti-fentanyl antibodies. Structures of some published fentanyl haptens are shown in Fig. 1b-d [21–24]. Different haptens produce different antibodies, which target different portions of the fentanyl molecule for binding. This could explain some of the observed differences in sensitivity and specificity among commercial brands of FTS [25].

In 2019, the CDC developed the Traceable Opioid Material Kits (TOMs Kit) containing standards of emerging opioids of concern, including over 210 fentanyl analogs, influenced in part by DEA Emerging Threat Reports [26]. Despite the existence of hundreds of fentanyl analogs and new ones emerging, exploration into FTS cross-reactivity with fentanyl analogs has been rather limited. A 2022 study assessed the limit of detection of BTNX Rapid Response test strips for 17 fentanyl analogs and compared their findings to the brand’s reports [14]. They could detect 14 screened compounds at or below 1000 ng/mL but were unable to detect 2 analogs at these concentrations reported by the manufacturer: Carfentanil and furanyl fentanyl. A 2021 study screened 28 fentanyl analogs on four brands of urinary fentanyl test strips (BTNX Rapid Response, One Step, Nal von Minden, and Rapid Self-Test) and found each brand could detect 21–24 of the fentanyl analogs screened including Carfentanil at 1000 ng/mL, a finding that conflicts with that of Ju et al. [14, 27]. A summary of existing literature regarding 37 unique fentanyl analogs is shown in Table 1. Much of the existing literature centers around BTNX Rapid Response strips, with no literature at the time of this report validating the use of DanceSafe FTS. The only information regarding the cross-reactivity of DanceSafe FTS with fentanyl analogs comes from their website, stating that their strips can detect Carfentanil and some other unnamed compounds but this provides no information regarding limits of detection [28]. The conflicting reports on the detectability of Carfentanil highlight a concern of some in the harm reduction community- that fentanyl test strips behave differently, even those from the same brand. If a distributor changes suppliers, FTS may be fabricated with antibodies produced from different fentanyl haptens, resulting in antibodies with differing sensitivities and specificities toward fentanyl, fentanyl analogs, or other cross-reacting compounds. This concern is exacerbated by the supply chain disruptions caused from the COVID-19 pandemic.

**Table 1:** Comparison of experimental results to literature reports

		Lowest detected concentration of 37 fentanyl analogs in literature (ng/mL)						
	Hayes et al		BTNX Inc	Park et al	Bergh et al			
	BTNX Rapid Response lots DOA2206394, DOA2204104	DanceSafe lots K2051226, K2021217	BTNX rapid response**	BTNX rapid response	BTNX rapid response	Rapid self-test	One step	Nal von Minden
Fentanyl	200	200	200	200	100	100	100	100
Acetyl fentanyl	200	2,000	150	100	100	100	100	100
Acryl fentanyl	2000	200	–	–	100	100	100	100
Alfentanil	Not detected*	20,000	–	Not detected^‡^	Not detected^†^	Not detected^†^	Not detected^†^	Not detected^†^
Benzodioxole fentanyl	2000	Not detected*	–	–	1000	1,000	Not detected^†^	1000
Butyryl fentanyl	200	200	700	100	100	100	100	100
Carfentanil	2000	Not detected*	1000	Not detected^‡^	1000	1000	Not detected^†^	1000
*cis-*3-methyl Fentanyl	2000	2000	–	–	1000	1000	1,000	1,000
Crotonyl fentanyl	2000	200	–	–	100	100	100	100
Cyclopropyl fentanyl	2000	200	–	–	100	1000	100	100
Despropionyl-*ortho*-fentanyl	Not detected*	Not detected*	–	–	Not detected^†^	Not detected^†^	Not detected^†^	Not detected^†^
Furanyl fentanyl	2000	20,000	500	Not detected^‡^	100	1000	100	100
*meta*-Fluorofentanyl	2000	200	–	–	1000	1000	100	1000
Methoxyacetyl fentanyl	200	2000	–	–	100	100	100	100
*N*-benzyl Furanyl norfentanyl	2000	20,000	–	–	1000	1000	100	1000
Norcarfentanil	Not detected*	Not detected*	–	–	Not detected^†^	Not detected^†^	Not detected^†^	Not detected^†^
Ocfentanil	200	20,000	250	–	100	100	10,000	100
*ortho*-Fluorofentanyl	2000	2000	–	–	100	1000	10,000	100
*para*-Chloroisobutyryl fentanyl	2000	Not detected*	–	1000	1000	1000	1,000	1000
*para*-fluoro Cyclopropyl benzyl fentanyl	–	–	–	–	100	1000	100	1000
*para*-Fluorobutyryl fentanyl (FBF)	2000	200	–	100	1000	1000	100	100
*para*-Fluorofentanyl	200	200	200	–	100	100	100	100
*para*-Fluoroisobutyryl fentanyl (FIBF)	2000	Not detected*	–	–	100	100	100	100
*para*-methoxy-Butyryl fentanyl	2000	Not detected*	–	200	1000	1000	10,000	1000
Remifentanil	Not detected*	Not detected*	70,000	–	10,000	10,000	Not detected^†^	10,000
Senecioylfentanyl	2000	20,000	–	–	100	1000	1000	100
Sufentanil	20,000	20,000	100,000	–	10,000	10,000	Not detected^†^	10,000
Tetrahydrofuran fentanyl	200	not detected*	–	–	100	100	100	100
Valeryl fentanyl	2000	20,000	700	500	1000	1000	10,000	100
4-ANPP	Not detected*	Not detected*	–	200	–	–	–	–
*α*-methyl Acetyl fentanyl	200	2000	–	500	–	–	–	–
*β*-methyl Fentanyl	2000	200	500	500	–	–	–	–
*N*-methyl Norfentanyl	2000	200	–	500	–	–	–	–
Norfentanyl	20,000	200	–	Not detected^‡^	–	–	–	–
*ortho*-methyl Acetyl fentanyl	2000	20,000	–	100	–	–	–	–
Phenyl fentanyl	2000	Not detected*	–	500	–	–	–	–
Thiofentanyl	2000	200	–	200	–	–	–	–

The lot numbers of FTS used in previous studies are not reported. Bergh et al. tested standards at 1000 ng/mL. If they were detected at that concentration, they were diluted to 100 ng/mL and retried. If they were not detected at 1000 ng/mL, they were screened again at 10,000 ng/mL

*At 20,000 ng/mL or less in water

**In urine

^‡^At 1,000 ng/mL or less in water

^†^At 10,000 ng/mL or less in water

In this study, 251 synthetic opioids were screened on FTS from two major brands for drug checking- BTNX Rapid Response and DanceSafe. The aims of this study are to (1) assess the ability of these brands of fentanyl test strips to detect a comprehensive set of fentanyl analogs and fentanyl-related compounds and (2) determine which structural moieties are crucial for FTS reactivity. By screening a large number of fentanyl analogs, we gain clearer understanding on the capabilities of FTS to detect specific analogs. We can further look for patterns in the chemical structures of the analogs and correlate those patterns to FTS results to determine which structural characteristics are most important for FTS detection, what changes in fentanyl structure inhibit detectability on FTS, and how these differ among brands.

## Materials and methods

### Reagents, chemicals, and supplies

BTNX Rapid Response™ fentanyl test strips (cut-off 20 ng/mL) lots DOA2206394 and DOA2204104 were purchased from Lochness Medical Supplies Inc. (Buffalo, NY, USA). DanceSafe branded fentanyl test strips (cut-off 10 ng/mL), manufactured by WHPM, lots K2051226 and K2021217 were purchased from DanceSafe (Albuquerque, NM, USA). Fentanyl test strips were run and analyzed according to manufacturer instructions. A Traceable Opioid Materials (TOMs) Fentanyl Analog Screening (FAS) kit and its four emergent panels containing 250 separate standards of fentanyl, fentanyl analogs, synthetic precursors, intermediates and impurities, and other synthetic opioids were received from Cayman Chemical Company (Ann Arbor, MI, USA). These standards were primarily in salt form, with residual glycerol and prepared according to manufacturer instructions, using 500 μL of HPLC-grade methanol to reconstitute as 400 μg/mL standard solutions. Certified reference material Carfentanil (100 μg/mL in methanol) was purchased from Cayman Chemical Company (Ann Arbor, MI, USA). HPLC-grade methanol was purchased from Azer Scientific (Morgantown, PA). All 250 FAS kit standards and Carfentanil were diluted to 20,000 ng/mL, 2000 ng/mL, 200 ng/mL, and 20 ng/mL concentrations in 18 ΜΩ deionized water.

### Compounds screened in this study

251 compounds were screened, including the contents of the TOMs Fentanyl Analog Screening (FAS) kit with emergent panels 1–4 and Carfentanil. Of these, 31 were non-fentanyl synthetic opioids; their structures are shown in Additional file 1: Fig. S1. Most (17) are “U” synthetic opioids, namely U-47700 which classified as a schedule 1 substance in 2016 after the DEA reported at least 46 fatalities associated with its use [29]. Three are benzimidazole-opioids, or nitazenes, (metonitazene, isotonitazene, etonitazene) which, like fentanyl, are μ-opioid agonists and had been reported in at least 94 toxicology reports by 2022 [30]. Five compounds feature piperazine moieties, compared to fentanyl’s piperidine, and similarly bind to the *μ*-opioid receptor [31]. Other notable synthetic opioids include brorphine, which emerged in the illicit drug market in 2019 and tianeptine, which is currently unscheduled but has been reported in forensic reports since 2018 [32, 33].

Six compounds in this study were synthetic precursors, intermediates, impurities, or metabolites of fentanyl and its analogs including 4-anilino-1-benzylpiperidine, 4-anilinopiperidine, 4-piperidone, N-benzyl-4-piperidone, NPP, and 4-ANPP. Their structures are shown in Additional file 1: Fig. S2.

The remaining 214 compounds were fentanyl or fentanyl analogs. Fentanyl analogs feature at least one modification to any of the moieties defined in Fig. 1A. For the sake of this analysis, the fentanyl molecule has been divided into 4 moieties which will be referred to as carbonyl, aniline, piperidine, or phenethyl and each carbon has been labeled with the notation used in naming analogs resulting from a modification to that carbon. The specific modifications in each compound are summarized in Additional file 1: Table S2. The haptens illustrated in Fig. 1B and C leave the carbonyl moiety more exposed for antibody recognition, while the hapten illustrated in Fig. 1D leaves the phenethyl group more exposed for antibody recognition.

### Lateral flow immunoassay fentanyl test strip analysis

Two lots of BTNX Rapid Response fentanyl test strips (DOA2206394 and DOA2204104, cut-off 20 ng/mL) and two lots of DanceSafe fentanyl test strips (K2051226 and K2021217, cut-off 10 ng/mL) were used in this study. Strips were immersed individually in the test solution for 30 s, then laid flat on a clean paper towel for 5 min to develop. A fentanyl test strip from each lot was tested with a positive control (20,000 ng/mL fentanyl in water) and negative control (100% water) and gave expected results. Stock solutions of fentanyl-related compounds were diluted to 20,000 ng/mL, 2000 ng/mL, 200 ng/mL, and 20 ng/mL in 100% water. Each compound was initially screened at 2000 ng/mL on 1 test strip from each lot (4 FTS total; 2 FTS from each brand). If a compound was detectable by a brand at 2000 ng/mL, it was then tested at 200 ng/mL. If it gave a positive at 200 ng/mL, it was tested at 20 ng/mL. If an analyte was not detectable at 2000 ng/mL, it was tested at 20,000 ng/mL.

FTS were analyzed by eye per manufacturer recommendations and scored as “pos”, “pos*”, “neg”, or “neg*.” A “pos” indicates that the reader is confident the test is positive when reading by eye while “neg” indicates that the reader is confident the test is negative when reading by eye. The asterisk denotes a lack of confidence by the reader in assessing the readout.

For a brand’s FTS to be classified as being able to detect a compound at a concentration, both lots must give a positive (pos or pos*) test at that concentration. If a FTS from one lot was positive, while the other was a negative, the brand is classified as unable to detect the compound at that concentration and its minimum detectable concentration is reported as the lowest concentration where FTS from *both* lots give a positive result. If the FTS from different lots gave different results at the highest tested concentration (20,000 ng/mL), the verdict is that the compound is not detectable with that brand and demarked with “ND*” in Additional file 1: Table S1.

### Testing in street drug samples

Street samples of cocaine HCl and heroin of unknown purity were obtained as discarded police seizures from the Berrien County Forensic Lab. Following DanceSafe drug checking recommendations, 35 mg of drug was dissolved in 3.5 mL of deionized water and separated into 7 aliquots of 0.5 mL. Cocaine HCl solutions were used as is, while each heroin solution was diluted with an additional 0.5 mL of water. To determine whether compounds could be detected by FTS in street drug sample matrixes, each aliquot was spiked with 2 uL of water, or 2 uL of 400 ug/mL fentanyl, tetrahydrofuran fentanyl, *α*’-methoxy fentanyl, N-(2C-D) fentanyl, N-(2C-G)-fentanyl, or N-(3,4,5-TMA) fentanyl to yield solutions at about 1600 ng/mL or 800 ng/mL of spiked compound for cocaine and heroin, respectively. These solutions were then tested with 1 FTS from each brand (BTNX lot DOA2204104 and DanceSafe lot K2051226) and read as “pos,” “pos*,” “neg*,” or “neg.”

### Data analysis

Of the 251 compounds screened on FTS, 217 were selected for further analysis to determine if specific structural features inhibit or permit FTS detection. Since FTS are designed to detect fentanyl, the chemical structure of each of these 217 compounds was compared to fentanyl, and the modifications occurring at each moiety (Fig. 1A) were tabulated. For each compound, the results of screening for both DanceSafe and BTNX FTS, the specific modifications made to each moiety, and the total number of moieties modified are given in Additional file 1: Table S2.

To determine if modifications to particular moieties impact detectability, all compounds that were not detectable by DanceSafe FTS were plotted in an UpSet plot using the UpSetR function in RStudio (Version 2022.12.0 + 353). The UpSet plot is used to visualize the combinations of moiety modifications present in the set of compounds. The combinations of moiety co-modifications are displayed in the bottom panel of the plot, while horizontal bars represent the frequency of modification to each moiety and the vertical bars represent the frequency of each combination of moiety modifications. In the UpSet plot, specific modifications are ignored and only the location of modification is assessed. An UpSet plot was also generated for compounds that were not detectable by BTNX FTS.

After assessing how the general location of modification impacts detectability, we sought to determine whether specific modifications predictably inhibit detection. Compounds that contained only one modification (with no co-modifications to other moieties of the molecule) were correlated with FTS screening results for both brands. If a compound with one modification was detectable by a brand, then that modification does not inhibit detection. If a compound with one modification was not detectable by a brand, then, the modification did inhibit detection. If there were multiple compounds containing that same modification (with other co-occurring modifications), results of FTS screening were cross-checked with that of the compound with the lone modification, to determine if that modification could reliably inhibit or allow detection. Through this method, specific modifications causing non-detection were identified.

## Results and discussion

### Comparison of BTNX and DanceSafe FTS results

**Fig. 2:**
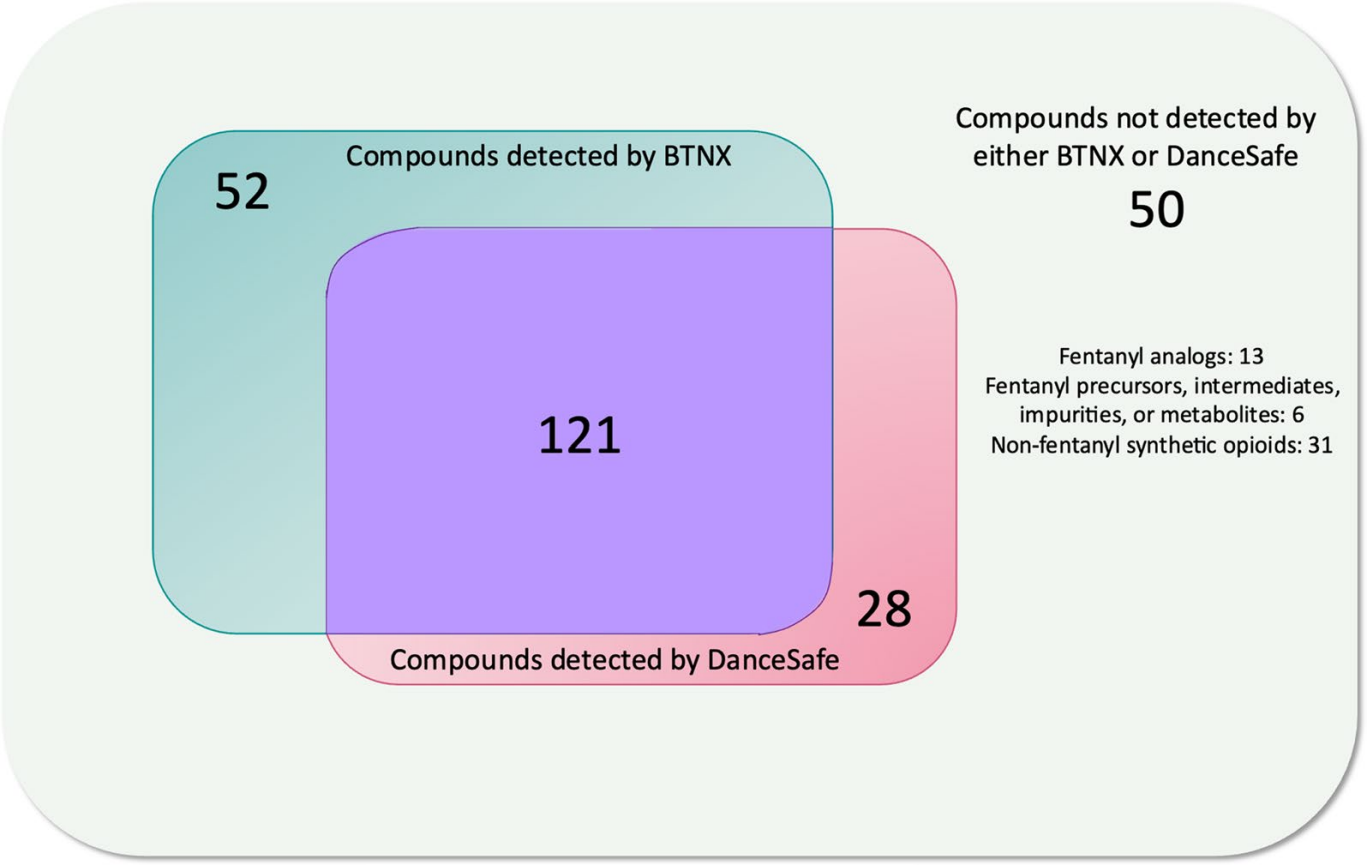
Summary of BTNX and DanceSafe detection of 251 screened compounds at 20,000 ng/mL

The experimental limit of detection for each type of test strip and each compound tested can be found in Additional file 1: Table S1. A summary of the findings is shown in Fig. 2. Neither brand detected any of the 31 non-fentanyl synthetic opioids (shown in Additional file 1: Fig. S1) or the 6 precursors, intermediates, metabolites, or impurities of fentanyl (analog) synthesis. The remaining 214 compounds were fentanyl or fentanyl analogs; 121 were detectable by both brands, 52 were detectable by BTNX but not DanceSafe, 28 were detectable by DanceSafe but not BTNX, and 13 were not detectable by either brand.

**Fig. 3:**
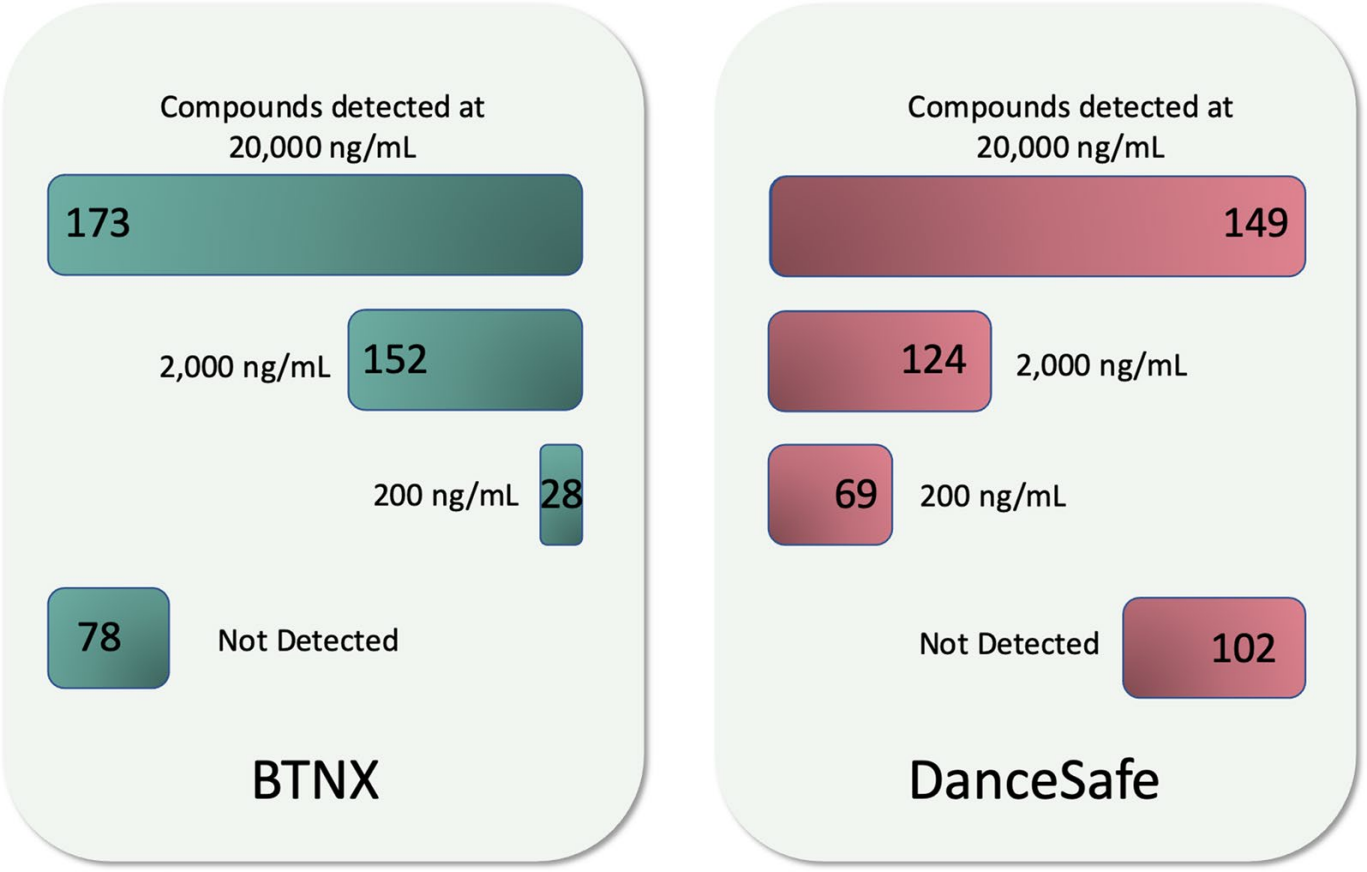
Sensitivity of BTNX and DanceSafe FTS to 251 screened compounds at 20,000, 2,000, and 200 ng/mL

Compounds were first tested at 2000 ng/mL. Depending on the results, they were then tested at 20,000 ng/mL or 200 ng/mL. Compounds positive at 200 ng/mL were also tested at 20 ng/mL, but no compounds were detectable by either brand at that concentration. A chart summarizing the number of compounds detected at each concentration is shown in Fig. 3. Fentanyl was detected by both brands at 200 ng/mL which is 10 times the stated cut-off of BTNX FTS for urinalysis, and 20 times the stated cut-off of DanceSafe FTS. This finding is in-line with previous reports of the LOD of BTNX strips, which found the working limit of detection for drug checking to be between 100 and 200 ng/mL [14–18]. We did not find previous literature reports of the limit of detection for DanceSafe FTS for drug checking applications.

While there is no standard sample preparation method for FTS checking, DanceSafe provides recommendations on their website. [28] For solid drugs, they recommend preparing a solution at a nominal concentration of 10 mg/mL of sample. The amount of fentanyl in a drug varies, but even if fentanyl was only present at 0.1% w/w of the sample, these preparation conditions would ensure the fentanyl is at a concentration of 10 μg/mL, well above the 200 ng/mL experimental cut-off concentration of fentanyl. For drugs prepared for intravenous use, they recommend diluting the residue left in the spoon or cooker—about 10 μL—with 1 mL of water. Typical doses of injectable opioids are around 10 mg/mL, so these preparation instructions ensure a concentration of 0.1 mg/mL of sample in water. If fentanyl was only 1% w/w of the starting drug, it would be at a final concentration of 1,000 ng/mL for testing, well above the 200 ng/mL experimental cut-off concentration of fentanyl.

BTNX FTS detected more compounds at or below 20,000 ng/mL than DanceSafe FTS (173 vs. 149) but DanceSafe FTS detected almost 2.5 times more compounds at 200 ng/mL than BTNX FTS (69 vs. 28). The results of this study generally agree with previous reports of BTNX FTS shown in Table 1. BTNX FTS were able to detect Carfentanil at 2000 ng/mL which is in-line with manufacturer and Bergh et al.’s reports of 1000 ng/mL but disagrees with the findings of Park et al. which did not detect Carfentanil at 1000 ng/mL. The most glaring discrepancy is with 4-ANPP which Park et al. report detecting at 200 ng/mL on BTNX FTS but was undetectable in this study at 20,000 ng/mL. Notably, the lots of FTS used in previous studies of fentanyl analog cross-reactivity were not reported but were almost certainly completed with different lots since previous experiments were done at least a year prior to this report. No literature reports of DanceSafe FTS cross-reactivity were available at time of publication.

### Overview of fentanyl analog modifications

The chemical structures of 217 compounds included in this study were systematically assessed to determine if certain structural features of fentanyl analogs reliably inhibit or permit FTS detectability. 34 compounds screened in this study were excluded from analysis including non-fentanyl synthetic opioids (*n* = 31) and synthetic impurities and metabolic products of fentanyl (*n* = 3); their structures are shown in Additional file 1: Fig. S1 and S2B. Three synthetic precursors, 4-anilinopiperidine, 4-anilino-1-benzylpiperidine, and 4-ANPP, were included due to their structural similarity to some fentanyl analogs. Their structures are shown in Additional file 1: Fig. S2A.

**Fig. 4:**
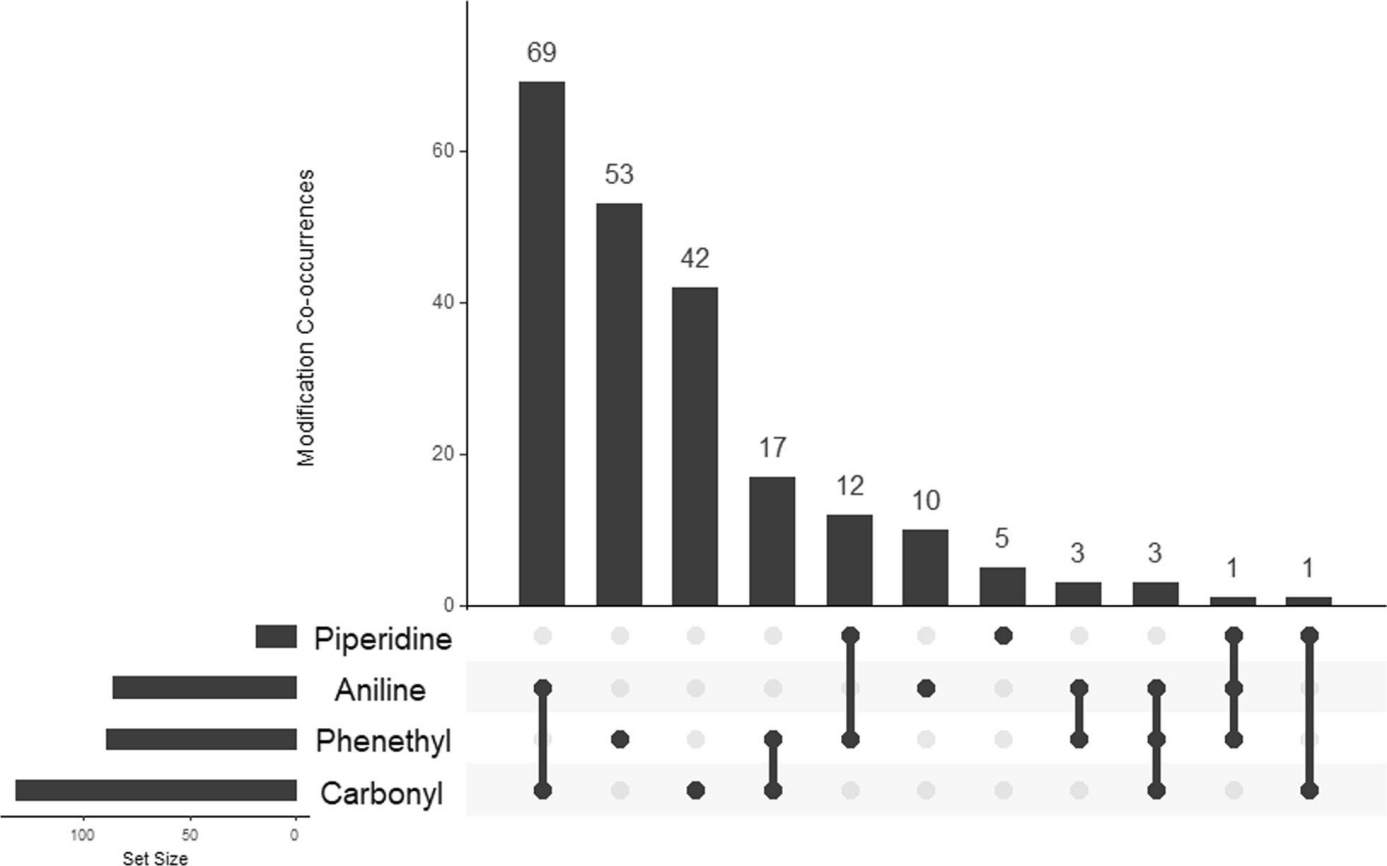
UpSet plot detailing co-occurrence of moiety modifications

Modifications may occur alone or in tandem with modifications at other moieties in the molecule. An UpSet plot detailing modification co-occurrences is shown in Fig. 4. Modifications occur most frequently at the carbonyl moiety and least frequently at the piperidine and occur at a single moiety (*n* = 110), co-occur with a modification at another moiety (*n* = 102), or co-occur with modifications at 2 different moieties (*n* = 4). Fentanyl itself has no modifications and is not included in this UpSet plot.

### Modifications & FTS detectability

**Fig. 5:**
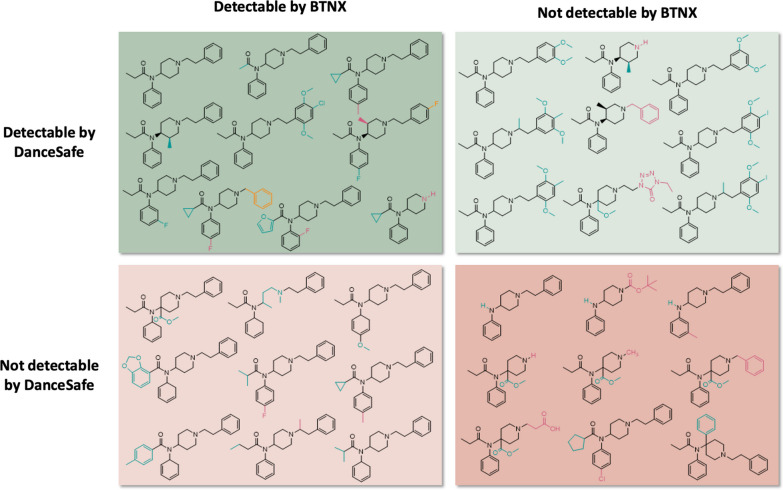
Structures of selected compounds detected and not detected by both brands

We hypothesize that differences in sensitivity and specificity between BTNX and DanceSafe FTS can be explained by a use of different antibodies on their test lines, probably generated by different haptens used in development. The specific modifications found in each fentanyl analog are listed in Additional file 1: Table S2. Modifications were correlated with results from both brands of FTS to determine which modifications inhibit FTS reactivity. Structures of select compounds detected and not detected by each brand are shown in Fig. 5. This analysis focuses on modifications that *alone* prevent detection, ie no other co-occurring modifications contribute to non-detection. Only two solo modifications cause non-detection for both BTNX and DanceSafe FTS; they are described in Additional file 1: Table S3. The first is a 4-phenyl substitution to the aniline ring which occurs in one compound, 4-phenyl fentanyl, with no other co-occurring modifications. The second is a modification to the carbonyl moiety, where the propionyl chain is replaced with a proton. This modification occurs in 7 compounds (Additional file 1: Table S3) including 4-ANPP where it is the lone modification, assuring us that the replacement of the carbonyl with a proton inhibits reactivity with both BTNX and DanceSafe FTS.

Modifications that alone are enough to inhibit detection for DanceSafe FTS but not BTNX are summarized in Additional file 1: Table S4. Some are present in multiple compounds including a 4-methyl acetate substitution to the piperidine ring (6 compounds), para-methoxy substitution to the aniline ring (8 compounds), and replacement of the carbonyl propionyl group with isobutyryl (6 compounds), tetrahydrofuran (4 compounds), or cyclopentyl (4 compounds). As shown in Additional file 1: Figure S3, majority of the compounds that are not detectable by DanceSafe FTS have modifications to the carbonyl group but not all modifications to the carbonyl moiety cause non-detection, shown in Additional file 1: Figure S4. In general, replacements with bulkier groups are more likely to inhibit DanceSafe FTS’s ability to detect the compound, though there are 5 membered rings that both inhibit and do not impact detection. Notably, there are no modifications to the phenethyl group that alone cause non-reactivity with DanceSafe FTS.

Modifications that inhibit detection for BTNX FTS are summarized in Additional file 1: Fig. S5. Interestingly, modifications to the phenethyl group are the only modifications that inhibit detection without any other co-occurring modifications. (Apart from the 4-phenyl aniline substitution and replacement of carbonyl propionyl chain with H, which causes non-detection for both brands). The structures of compounds with only modifications to the phenethyl region which are non-detectable by BTNX FTS are shown in Additional file 1: Fig. S6. However, not all modifications to the phenethyl group inhibit detection, as shown in Additional file 1: Fig. S7. In general, bulkier modifications inhibit detection. Despite being present in some of the non-detected compounds, alkyl or hydroxy substitutions to the *α*- and *β*- carbons of the phenethyl chain and single alkyl, methoxy, or halogen substitutions to the phenethyl ring do not inhibit detection on their own. Replacement of the phenyl group with a proton, methyl, benzyl, or other heterocycle also does not prevent detection. The majority of the non-detected compounds have dimethoxy substitutions to the 2’ and 5’ carbons of the phenethyl ring with additional substitutions of alkyl or halogen groups to the 4’ position. Interestingly, two compounds- N-(2C-C)-fentanyl and N-(2C-B)-fentanyl- fit this description but *are* detectable by BTNX FTS, while similar compounds like N-(2C-I)-fentanyl and N-(DOB)-fentanyl and N-(DOC)-fentanyl are *not* detectable by BTNX FTS; their structures are shown in Additional file 1: Fig. S8. Iodine is larger than chlorine or bromine which could explain N-(2C-I)-fentanyl’s inability to be detected, while the additional *α*-methyl substitutions—while not bulky enough to prevent detection on their own—could make the phenethyl groups large enough inhibit detection of N-(DOB)-fentanyl and N-(DOC)-fentanyl.

The antibodies used on these two FTS products recognize different sides of the fentanyl molecule. In general, DanceSafe FTS are most sensitive to modifications to the carbonyl moiety and resistant to modifications to the phenethyl group, while BTNX FTS are most sensitive to the phenethyl moiety and are resistant to modifications to the carbonyl moiety—except for replacement of the carbonyl with a proton. Modifications to these respective moieties do not guarantee inhibition, though bulkier groups are more likely to prevent detection. The manufacturer of the DanceSafe FTS confirmed that their hapten is bound via the piperidine group, similar to the hapten shown in Fig. 1C. During antigen generation, this hapten exposes the carbonyl end of the fentanyl molecule to the immune system, so the resulting antibodies are able to reject fentanyl analogs which contain modifications of the carbonyl moiety. The manufacturer of the BTNX FTS confirmed that their hapten is bound via the carbonyl group, similar to the hapten shown in Fig. 1D. During antibody generation, this hapten exposes the phenethyl end of the fentanyl molecule to the immune system, so the resulting antibodies are able to reject fentanyl analogs that contain modifications to the phenethyl moiety.

### Different FTS specificities could be a tool for harm reduction

Due to differences in fentanyl analog sensitivity and specificity between brands, utilizing both FTS brands could be a useful drug checking strategy. DanceSafe FTS could be used to detect compounds in BTNX’s “blind spot” and vice versa. To determine the viability of this method, street samples of cocaine HCl and heroin were prepared according to DanceSafe’s drug checking instructions and spiked with water, fentanyl, tetrahydrofuran fentanyl, *α′*-methoxy fentanyl, N-(2C-D) fentanyl, N-(2C-G)-fentanyl, or N-(3,4,5-TMA) fentanyl, so the solutions had a final concentration of about 1600 ng/mL of spiked compound. Heroin solutions had to be diluted by half, as an unspiked control at the original concentration gave a positive result on BTNX FTS. Likely, this street sample of heroin contained the common cutting agent diphenhydramine, which is known to cause false positives on BTNX FTS [19]. Diluting the solution gave positive and negative controls as expected. The fentanyl analogs used in this experiment were selected because they were detectable in standard solutions at 200 ng/mL by at least one brand. Standard solutions of tetrahydrofuran fentanyl and α’- methoxy fentanyl were detectable by BTNX but not by DanceSafe FTS, while standard solutions of N-(2C-D) fentanyl, N-(2C-G) fentanyl, and N-(3,4,5-TMA) fentanyl were detectable by DanceSafe FTS but not BTNX FTS. Results of this experiment are shown in Table 2 and show similar results to standard solutions. Though the test lines produced by N-(2C-D) fentanyl and N-(2C-G) fentanyl on BTNX FTS were very faint in the heroin solutions, they were clear in the cocaine solutions. Using both brands in drug checking could alert a user to fentanyl analogs in the “blind spot” of one of the brands.

**Table 2:** Differing reactivity of BTNX (blue) and DanceSafe (yellow with black “FEN”) FTS in street drug samples spiked with fentanyl analogs

Spiked compound	Standard solution		Cocaine			Heroin		
	DS	BTNX	FTS images	DS	BTNX	FTS images	DS	BTNX
Water	neg	neg		neg	neg		neg	neg
Fentanyl	pos	pos		pos	pos		pos	pos
Tetrahydrofuran fentanyl	neg	pos		neg	pos		neg	pos
*α*′-methoxy fentanyl	neg	pos		neg	pos		neg	pos
*N*-(2C-D) fentanyl	pos	neg		pos	neg		pos	neg*
*N*-(2C-G) fentanyl	pos	neg		pos	neg		pos	neg*
*N*-(3,4,5-TMA) fentanyl	pos	neg		pos	neg		pos	neg

Two results denoted by neg* were rated as weak negatives by the reader

## Conclusions

Understanding the limitations and applications of FTS brands could have implications for drug checking. There were 52 fentanyl analogs in the blind spot of DanceSafe FTS and 28 fentanyl analogs in the blind spot of BTNX FTS, meaning one third of all fentanyl analogs screened in this study are detectable by one brand but not the other. Utilizing both brands of FTS could help ensure fentanyl analogs are not missed in screening. Further, if a street sample is tested and one brand gives a positive while another gives a negative that could be an indication that further non-targeted analysis of the sample needs to be performed to identify if a new or uncommon fentanyl analog is present in the sample.

In this study, BTNX FTS detected 173 compounds compared to DanceSafe FTS that detected 149 compounds. DanceSafe FTS are much more sensitive to bulky modifications to the carbonyl region while BTNX FTS are more sensitive to bulky substitutions at the phenethyl moiety. Notably more fentanyl analogs in this study had carbonyl modifications than phenethyl modifications. While BTNX FTS detected more compounds, DanceSafe FTS had relatively higher sensitivity toward the compounds they could detect, detecting almost 2.5 times more compounds at 200 ng/mL than BTNX FTS. The differences in activity of these FTS are likely due to different test antibodies resulting from different haptens used in antibody production.

While FTS of different lots within the same brand had similar reactivities in this study, the FTS were purchased within a relatively short timeframe making it unlikely that any changes in supplier or production had occurred. If FTS distributors change antibody  that  is likely to change the sensitivity and specificity of their products and could account for the differences between this study and previous reports from Park et al. [14] and Bergh et al. [27] which have disagreement on the detectability of 4-ANPP by BTNX test strips. As a policy, FTS manufacturers should report what antibodies they are using or at least when they change antibodies, so FTS users and researchers can be aware that sensitivity toward select fentanyl analogs may be affected. Because FTS for drug checking are not currently regulated by any government agencies in the US, using both brands of FTS could be a safeguard against lot-to-lot and brand-to-brand variability.

### Supplementary Information

**Additional file 1:** This file contains supporting information, including the limits of detection, structures, and modifications of all compounds examined in this study and additional UpSet plots illustrating co-modifications.

## Data Availability

All data generated or analyzed during this study are included in this published article and its supplementary information files.
