# Supplementary material for: Assessment of two brands of fentanyl test strips with 251 synthetic opioids reveals “blind spots” in detection capabilities
Source: Harm Reduct J. 2023 Dec 6;20:175. doi: 10.1186/s12954-023-00911-w (PMC10702106; doi:10.1186/s12954-023-00911-w)

**Assessment of two brands of fentanyl test strips with 251 synthetic opioids reveals “blind spots” in detection capabilities**

Kathleen L. Hayes, Marya Lieberman

**Supporting Information**

Table S1: limit of detection for 251 compounds screened in this study 2

Figure S1: structures of non-fentanyl synthetic opioids excluded from structure analysis 8

Figure S2: Structures of synthetic precursors, intermediates, and impurities included in structure study (A) and excluded from structure study (B) 9

Table S2: 217 fentanyl analogs and fentanyl-related compounds included in structure study and their specific modifications 10

Table S3: modifications that cause non-detection for both BTNX and DanceSafe FTS 21

Table S4: modifications that cause non-detection for DanceSafe FTS without any co-occurring modifications 21

Figure S3: UpSet plot of all compounds that are not detectable by DanceSafe FTS 25

Figure S4: Carbonyl modifications that do NOT cause non-detects for DanceSafe (on their own, without any co-occurring modifications) 26

Figure S5: UpSet plot of co-modifications that cause non-detection for BTNX FTS (including compounds ND by DanceSafe as well) 27

Figure S6: Phenethyl modifications that inhibit BTNX detection 28

Figure S7: Phenethyl modifications that do not inhibit BTNX detection 30

Figure S8: left: N-(2C-B) Fentanyl (top) and N-(2C-C) Fentanyl (bottom) are detectable by BTNX FTS; right: structurally similar compounds N-(DOB) fentanyl (top) and N-(DOC) fentanyl (bottom) and N-(2C-I) Fentanyl (right) are not detectable by BTNX FTS 31

# **Table S1:** limit of detection for 251 compounds screened in this study

| Compound | BTNX limit of detection (ng/mL) | DanceSafe limit of detection (ng/mL) |
| --- | --- | --- |
| **Fentanyl analogs** | | |
| (±)-cis-3-methyl Butyryl fentanyl | 2,000 | 20,000 |
| (±)-cis-3-methyl Fentanyl | 2,000 | 2,000 |
| (±)-cis-3-methyl Norfentanyl | ND | 200 |
| (±)-cis-3-methyl Thiofentanyl | 2,000 | 2,000 |
| (±)-cis-Isofentanyl | ND | 2,000 |
| (±)-trans-3-methyl Fentanyl | 2,000 | 200 |
| (±)-trans-3-methyl Thiofentanyl | 2,000 | 200 |
| 2,2,3,3-tetramethyl-Cyclopropyl fentanyl | 20,000 | ND |
| 2,3-Benzodioxole fentanyl | 2,000 | ND |
| 2',3'-dimethoxy Fentanyl | 2,000 | 200 |
| 2,3-seco-Fentanyl | 20,000 | ND |
| 2',4'-dimethoxy Fentanyl | ND* | 200 |
| 2',5'-dimethoxy Fentanyl | 20,000 | 200 |
| 2',6'-dimethoxy Fentanyl | 20,000 | 200 |
| 2'-fluoro ortho-Fluorofentanyl | 2,000 | 2,000 |
| 2'-Fluorofentanyl | 2,000 | 200 |
| 2'-methyl Acetyl fentanyl | 200 | 2,000 |
| 2'-methyl Fentanyl | 2,000 | 200 |
| 3',4'-dimethoxy Fentanyl | ND | 200 |
| 3',5'-dimethoxy Fentanyl | ND | 2,000 |
| 3'-fluoro ortho-Fluorofentanyl | 2,000 | 200 |
| 3'-Fluorofentanyl | 2,000 | 200 |
| 3'-methyl Acetyl fentanyl | 200 | 2,000 |
| 3'-methyl Fentanyl | 2,000 | 200 |
| 4-Anilino-1-Boc-piperidine | ND | ND |
| 4'-fluoro, para-fluoro (±)-trans-3-methyl Fentanyl | 2,000 | 2,000 |
| 4'-Fluorofentanyl | 2,000 | 200 |
| 4'-methyl Acetyl fentanyl | 200 | 2,000 |
| 4'-methyl Fentanyl | 2,000 | 200 |
| 4-methyl Fentanyl | 2,000 | 2,000 |
| 4-Phenyl fentanyl | ND* | ND |
| Acetyl fentanyl | 200 | 2,000 |
| Acetyl norfentanyl | 20,000 | 20,000 |
| Acrylfentanyl | 2,000 | 200 |
| Alfentanil | ND | 20,000 |
| Benzodioxole fentanyl | 2,000 | ND |
| Benzyl Acrylfentanyl | 2,000 | 2,000 |
| Benzyl Carfentanil | ND | ND |
| Benzyl fentanyl | 2,000 | 200 |
| Butyryl fentanyl | 200 | 200 |
| Butyryl norfentanyl | 20,000 | 200 |
| Carfentanil | 2,000 | ND |
| Crotonyl fentanyl | 2,000 | 200 |
| Cyclobutyl fentanyl | 2,000 | 200 |
| Cyclohexyl fentanyl | 2,000 | ND |
| Cyclopentenyl fentanyl | 2,000 | 20,000 |
| Cyclopentyl fentanyl | 2,000 | ND |
| Cyclopropaneacetyl fentanyl | 2,000 | 2,000 |
| Cyclopropyl fentanyl | 2,000 | 200 |
| Despropionyl 2'-fluoro ortho-Fluorofentanyl | ND | ND |
| Despropionyl meta-Methylfentanyl | ND | ND |
| Despropionyl ortho-Fluorofentanyl | ND | ND |
| Despropionyl para-Fluorofentanyl | ND | ND |
| Ethoxyacetyl fentanyl | 200 | ND* |
| Fentanyl | 200 | 200 |
| Fentanyl Carbamate | 2,000 | 200 |
| Fentanyl Methyl Carbamate | 2,000 | 200 |
| FIBF | 2,000 | ND |
| Furanyl fentanyl | 2,000 | 20,000 |
| Furanyl fentanyl 3-furancarboxamide isomer | 2,000 | 200 |
| Furanyl norfentanyl | 20,000 | 2,000 |
| Furanylethyl fentanyl | 2,000 | 200 |
| Heptanoyl fentanyl | 20,000 | ND |
| Hexanoyl fentanyl | 20,000 | ND |
| Isobutyryl fentanyl | 2,000 | ND |
| Isovaleryl fentanyl | 2,000 | ND |
| meta-fluoro Acrylfentanyl | 2,000 | 2,000 |
| meta-fluoro Furanyl fentanyl | 2,000 | 2,000 |
| meta-fluoro Methoxyacetyl fentanyl | 200 | 2,000 |
| meta-fluoro Valeryl fentanyl | 2,000 | ND |
| meta-Fluorobutyryl fentanyl | 2,000 | 2,000 |
| meta-Fluorofentanyl | 2,000 | 200 |
| meta-Fluoroisobutyryl fentanyl | 2,000 | ND |
| meta-methoxy Furanyl fentanyl | 2,000 | ND* |
| meta-methyl Acetyl fentanyl | 200 | 20,000 |
| meta-methyl Cyclopropyl fentanyl | 2,000 | 200 |
| meta-methyl Furanyl fentanyl | 2,000 | 20,000 |
| meta-methyl Methoxyacetyl fentanyl | 200 | 20,000 |
| meta-Methylfentanyl | 2,000 | 2,000 |
| Methacrylfentanyl | 2,000 | 20,000 |
| Methoxyacetyl fentanyl | 200 | 2,000 |
| N-(2,5-DMA) Fentanyl | 2,000 | 200 |
| N-(2-APB) Fentanyl | 20,000 | 200 |
| N-(2C-B) Fentanyl | 20,000 | 2,000 |
| N-(2C-B-fly) Fentanyl | ND | 2,000 |
| N-(2C-C) Fentanyl | 20,000 | 2,000 |
| N-(2C-D) Fentanyl | ND | 200 |
| N-(2C-E) Fentanyl | ND | 2,000 |
| N-(2C-G) Fentanyl | ND | 200 |
| N-(2C-I) Fentanyl | ND* | 2,000 |
| N-(2C-iP) Fentanyl | ND | 2,000 |
| N-(2C-N) Fentanyl | ND | 200 |
| N-(2C-P) Fentanyl | ND | 20,000 |
| N-(2C-T) Fentanyl | ND | 200 |
| N-(2C-T-2) Fentanyl | ND | 200 |
| N-(2C-T-4) Fentanyl | ND | 2,000 |
| N-(2C-T-7) Fentanyl | ND | 2,000 |
| N-(2C-TFM) Fentanyl | ND | 2,000 |
| N-(3,4,5-TMA) Fentanyl | ND | 200 |
| N-(3C-B-fly) Fentanyl | ND | 200 |
| N-(3-ethylindole) Norfentanyl | 2,000 | 200 |
| N-(6-APB) Fentanyl | 2,000 | 200 |
| N-(6-APDB) Fentanyl | 2,000 | 200 |
| N-(DOB) Fentanyl | ND | 2,000 |
| N-(DOBU) Fentanyl | ND | 2,000 |
| N-(DOC) Fentanyl | ND | 200 |
| N-(DOET) Fentanyl | ND | 2,000 |
| N-(DOI) Fentanyl | ND | 2,000 |
| N-(DOM) Fentanyl | ND | 200 |
| N-(MDA) Fentanyl | 2,000 | 200 |
| N-(Phentermine) Fentanyl | 2,000 | 200 |
| N,N-Dimethylamido-despropionyl fentanyl | 200 | 2,000 |
| N-benzyl Furanyl norfentanyl | 2,000 | 20,000 |
| N-benzyl para-fluoro Cyclopropyl norfentanyl | 2,000 | 2,000 |
| N-benzyl para-fluoro Norfentanyl | 2,000 | 200 |
| N-Benzyl phenyl norfentanyl | 2,000 | ND |
| N-methyl Cyclopropyl norfentanyl | 2,000 | 200 |
| N-methyl Norcarfentanil | ND | ND |
| N-methyl Norfentanyl | 2,000 | 200 |
| Norcarfentanil | ND | ND |
| Norfentanyl | 20,000 | 200 |
| Norsufentanil | ND | 2,000 |
| Ocfentanil | 200 | 20,000 |
| ortho-fluoro Acrylfentanyl | 2,000 | 2,000 |
| ortho-fluoro Furanyl fentanyl | 2,000 | 20,000 |
| ortho-fluoro Valeryl fentanyl | 2,000 | ND |
| ortho-Fluorobutyryl fentanyl | 2,000 | 2,000 |
| ortho-Fluorofentanyl | 2,000 | 2,000 |
| ortho-Fluoroisobutyryl fentanyl | 2,000 | ND |
| ortho-isopropyl Furanyl fentanyl | 20,000 | ND |
| ortho-methoxy Butyryl fentanyl | 2,000 | ND* |
| ortho-methoxy Furanyl fentanyl | 2,000 | ND* |
| ortho-methyl Acetyl fentanyl | 2,000 | 20,000 |
| ortho-methyl Acrylfentanyl | 2,000 | 2,000 |
| ortho-methyl Cyclopropyl fentanyl | 2,000 | 200 |
| ortho-methyl Furanyl fentanyl | 2,000 | 20,000 |
| ortho-methyl Methoxyacetyl fentanyl | 200 | 20,000 |
| ortho-methyl Phenyl fentanyl | 2,000 | ND |
| ortho-Methylfentanyl | 2,000 | 200 |
| para-Bromofentanyl | 2,000 | 200 |
| para-chloro Acrylfentanyl | 2,000 | 200 |
| para-chloro Cyclobutyl fentanyl | 2,000 | 2,000 |
| para-chloro Cyclopentyl fentanyl | ND* | ND |
| para-chloro Cyclopropyl fentanyl | 2,000 | 2,000 |
| para-chloro Furanyl fentanyl | 2,000 | 20,000 |
| para-chloro Furanyl fentanyl 3-furancarboxamide | 20,000 | 2,000 |
| para-chloro Methoxyacetyl fentanyl | 2,000 | 2,000 |
| para-chloro Valeryl fentanyl | 20,000 | ND* |
| para-Chloroacetyl fentanyl | 200 | 2,000 |
| para-Chlorobutyryl fentanyl | 2,000 | 200 |
| para-Chlorofentanyl | 2,000 | 200 |
| para-Chloroisobutyryl fentanyl | 2,000 | ND* |
| para-fluoro 4-ANBP | ND | ND |
| para-fluoro Acrylfentanyl | 2,000 | 2,000 |
| para-fluoro Crotonyl fentanyl | 2,000 | 200 |
| para-fluoro Cyclopentyl fentanyl | 2,000 | ND |
| para-fluoro Cyclopropyl fentanyl | 2,000 | 200 |
| para-fluoro Furanyl fentanyl | 2,000 | 2,000 |
| para-fluoro Furanyl fentanyl 3-furancarboxamide | 2,000 | 200 |
| para-fluoro Methoxyacetyl fentanyl | 200 | 2,000 |
| para-fluoro Tetrahydrofuran fentanyl | 200 | ND |
| para-fluoro Valeryl fentanyl | 2,000 | ND* |
| para-Fluoroacetyl fentanyl | 200 | 2,000 |
| para-Fluorobutyryl fentanyl | 2,000 | 200 |
| para-Fluorofentanyl | 200 | 200 |
| para-hydroxy Butyryl fentanyl | 2,000 | 200 |
| para-methoxy Acetyl fentanyl | 2,000 | ND |
| para-methoxy Acrylfentanyl | 2,000 | ND |
| para-methoxy Butyryl fentanyl | 2,000 | ND |
| para-methoxy Furanyl fentanyl | 2,000 | ND |
| para-methoxy Methoxyacetyl fentanyl | 2,000 | ND |
| para-methoxy Tetrahydrofuran fentanyl | 2,000 | ND |
| para-methoxy Valeryl fentanyl | 20,000 | ND |
| para-Methoxyfentanyl | 2,000 | ND* |
| para-methyl Acetyl fentanyl | 200 | 20,000 |
| para-methyl Acrylfentanyl | 2,000 | 20,000 |
| para-methyl Butyryl fentanyl | 2,000 | 20,000 |
| para-methyl Cyclopentyl fentanyl | 20,000 | ND |
| para-methyl Cyclopropyl fentanyl | 2,000 | 20,000 |
| para-methyl Furanyl fentanyl | 2,000 | ND* |
| para-methyl Isobutyryl fentanyl | 2,000 | ND |
| para-methyl Methoxyacetyl fentanyl | 200 | 20,000 |
| para-methyl Tetrahydrofuran fentanyl | 2,000 | ND |
| para-Methylfentanyl | 2,000 | 2,000 |
| para-Toluoyl fentanyl | 20,000 | ND |
| Phenoxyacetyl fentanyl | 2,000 | ND |
| Phenyl fentanyl | 2,000 | ND |
| Phenylacetyl fentanyl | 2,000 | ND |
| Pivaloyl fentanyl | 2,000 | ND |
| Remifentanil | ND | ND |
| Remifentanil Acid | ND | ND |
| Senecioylfentanyl | 2,000 | 20,000 |
| Sufentanil | 20,000 | 20,000 |
| Tetrahydrofuran fentanyl | 200 | ND |
| Tetrahydrofuran fentanyl 3-tetrahydrofurancarboxamide | 200 | 2,000 |
| Tetrahydrothiophene fentanyl | 2,000 | ND |
| Thienyl fentanyl | 2,000 | 200 |
| Thiofentanyl | 2,000 | 200 |
| Thiophene fentanyl | 2,000 | 2,000 |
| Thiophene fentanyl 3-thiophenecarboxamide | 2,000 | ND* |
| Tigloyl fentanyl | 2,000 | ND |
| Valeryl fentanyl | 2,000 | 20,000 |
| α'-methoxy Fentanyl | 200 | ND |
| α-methyl Acetyl fentanyl | 200 | 2,000 |
| α'-methyl Butyryl fentanyl | 2,000 | ND |
| α-methyl Butyryl fentanyl | 2,000 | 200 |
| α-methyl Fentanyl | 2,000 | 200 |
| α-methyl Thiofentanyl | 2,000 | 200 |
| β-hydroxy Fentanyl | 200 | 200 |
| β-Hydroxythioacetylfentanyl | 200 | 2,000 |
| β-Hydroxythiofentanyl | 200 | 200 |
| β-methyl Acetyl fentanyl | 2,000 | 2,000 |
| β-methyl Fentanyl | 2,000 | 200 |
| β'-Phenyl fentanyl | 20,000 | ND |
| **Synthetic precursors, intermediates, impurities** | | |
| 4-Anilino-1-benzylpiperidine | ND | ND |
| 4-Anilinopiperidine | ND | ND |
| 4-Piperidone | ND | ND^†^ |
| N-Benzyl-4-piperidone | ND | ND^†^ |
| NPP | ND | ND^†^ |
| 4-ANPP | ND | ND |
| **Non-fentanyl synthetic opioids** | | |
| 2-fluoro MT-45 | ND* | ND |
| 2-fluoro Viminol | ND^†^ | ND |
| 2-methyl AP-237 | ND^†^ | ND |
| 3,4-Ethylenedioxy U-47700 | ND^†^ | ND |
| 3,4-Ethylenedioxy U-51754 | ND^†^ | ND |
| 3,4-Methylenedioxy U-47700 | ND^†^ | ND |
| 4-phenyl U-51754 | ND | ND |
| AH 7921 | ND | ND |
| AP-237 | ND^†^ | ND^†^ |
| AP-238 | ND^†^ | ND^†^ |
| Brorphine | ND^†^ | ND^†^ |
| Etonitazene | ND^†^ | ND |
| Isopropyl U-47700 | ND | ND |
| Isotonitazene | ND^†^ | ND |
| Metonitazene | ND^†^ | ND |
| MT-45 | ND | ND |
| N,N-didesmethyl U-47700 | ND^†^ | ND |
| N-desmethyl U-47700 | ND^†^ | ND |
| N-methyl U-47931E | ND^†^ | ND |
| O-Desmethyl-cis-tramadol | ND^†^ | ND |
| Piperidylthiambutene | ND^†^ | ND |
| Propyl U-47700 | ND^†^ | ND |
| Tianeptine | ND^†^ | ND |
| U-47700 | ND^†^ | ND |
| U-47931E | ND^†^ | ND |
| U-48520 | ND | ND |
| U-48753E | ND^†^ | ND |
| U-48800 | ND^†^ | ND |
| U-49900 | ND^†^ | ND |
| U-50488 | ND^†^ | ND |
| U-51754 | ND^†^ | ND |

* denotes lots gave discordant results; ^†^ denotes only 1 lot from each brand was tested, due to low supply

# **Figure S1:** structures of non-fentanyl synthetic opioids excluded from structure analysis


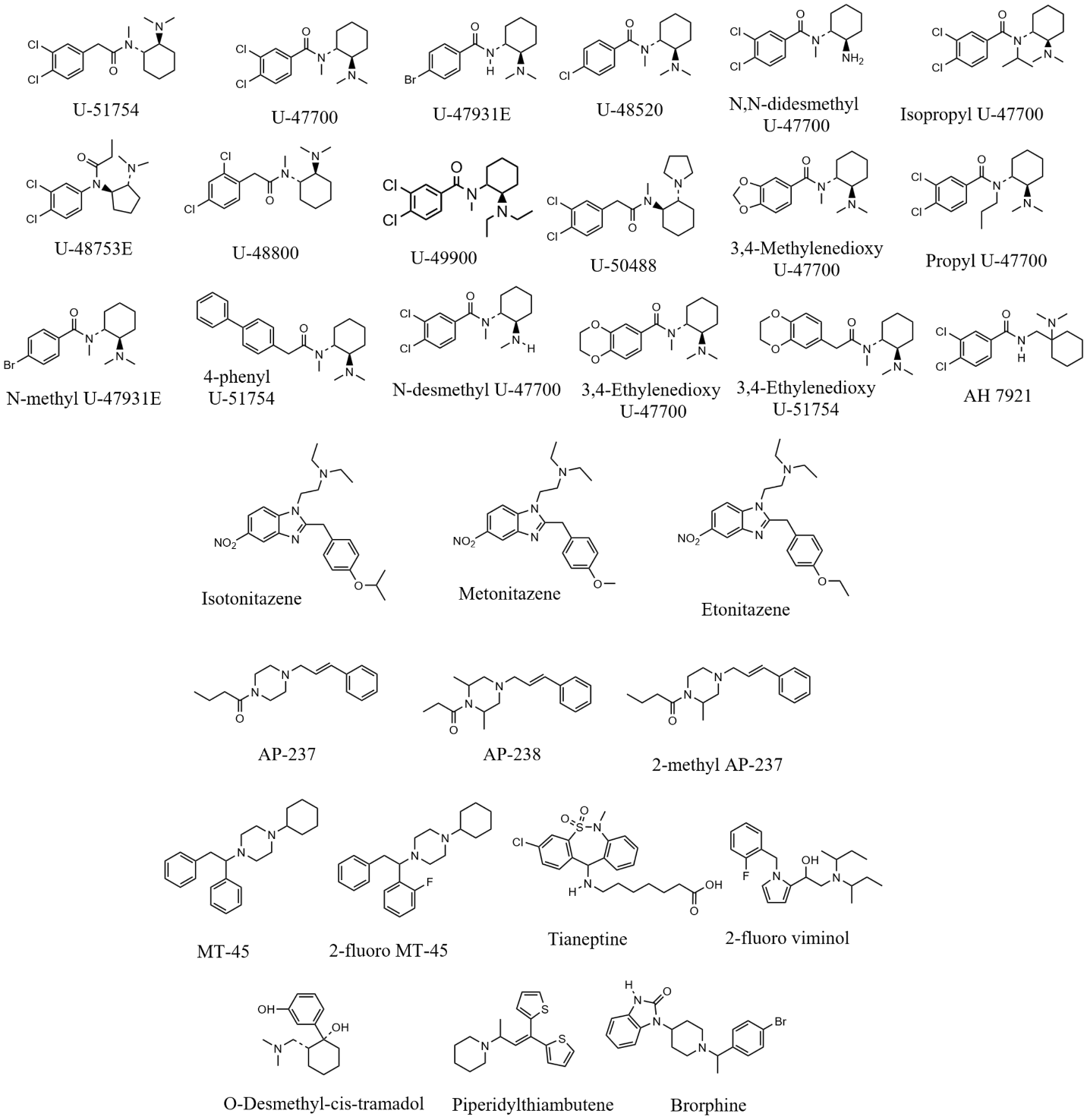


# **Figure S2**: Structures of synthetic precursors, intermediates, and impurities included in structure study (A) and excluded from structure study (B)

**
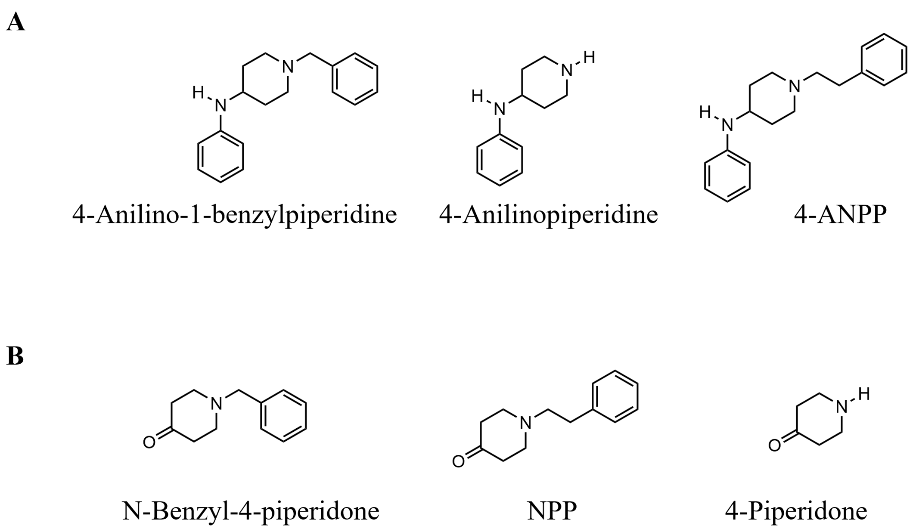
**

# **Table S2**: 217 fentanyl analogs and fentanyl-related compounds included in structure study and their specific modifications

| **Compound** | **BTNX LOD (ng/mL)** | **DanceSafe LOD (ng/mL)** | **Phenethyl** | **Piperidine** | **Aniline** | **Carbonyl** | **# mods** |
| --- | --- | --- | --- | --- | --- | --- | --- |
| 2,2,3,3-tetramethyl-Cyclopropyl fentanyl | 20,000 | ND |  |  |  | 2,2,3,3-tetramethyl cyclopropyl replacement | 1 |
| 2,3-Benzodioxole fentanyl | 2,000 | ND |  |  |  | 2,3-benzodioxole substitution | 1 |
| Furanyl fentanyl 3-furancarboxamide isomer | 2,000 | 200 |  |  |  | 3-furancarboxamide replacement | 1 |
| para-chloro Furanyl fentanyl 3-furancarboxamide | 20,000 | 2,000 |  |  | para-chloro substitution | 3-furancarboxamide replacement | 2 |
| para-fluoro Furanyl fentanyl 3-furancarboxamide | 2,000 | 200 |  |  | para-fluoro substitution | 3-furancarboxamide replacement | 2 |
| Tetrahydrofuran fentanyl 3-tetrahydrofurancarboxamide | 200 | 2,000 |  |  |  | 3-tetrahydrofurancarboxamide replacement | 1 |
| Thiophene fentanyl 3-thiophenecarboxamide | 2,000 | ND* |  |  |  | 3-thiophene replacemet | 1 |
| 2'-methyl Acetyl fentanyl | 200 | 2,000 | 2'-methyl substitution |  |  | acetyl replacement | 2 |
| 3'-methyl Acetyl fentanyl | 200 | 2,000 | 3'-methyl substitution |  |  | acetyl replacement | 2 |
| 4'-methyl Acetyl fentanyl | 200 | 2,000 | 4'-methyl substitution |  |  | acetyl replacement | 2 |
| Acetyl norfentanyl | 20,000 | 20,000 | H replacement |  |  | acetyl replacement | 2 |
| β-Hydroxythioacetylfentanyl | 200 | 2,000 | thio replacement; β-Hydroxy substitution |  |  | acetyl replacement | 2 |
| α-methyl Acetyl fentanyl | 200 | 2,000 | α-methyl substitution |  |  | acetyl replacement | 2 |
| β-methyl Acetyl fentanyl | 2,000 | 2,000 | β-methyl substitution |  |  | acetyl replacement | 2 |
| Acetyl fentanyl | 200 | 2,000 |  |  |  | acetyl replacement | 1 |
| meta-methyl Acetyl fentanyl | 200 | 20,000 |  |  | meta-methyl substitution | acetyl replacement | 2 |
| ortho-methyl Acetyl fentanyl | 2,000 | 20,000 |  |  | ortho-methyl substitution | acetyl replacement | 2 |
| para-Chloroacetyl fentanyl | 200 | 2,000 |  |  | para-chloro substitution | acetyl replacement | 2 |
| para-Fluoroacetyl fentanyl | 200 | 2,000 |  |  | para-fluoro substitution | acetyl replacement | 2 |
| para-methoxy Acetyl fentanyl | 2,000 | ND |  |  | para-methoxy substitution | acetyl replacement | 2 |
| para-methyl Acetyl fentanyl | 200 | 20,000 |  |  | para-methyl substitution | acetyl replacement | 2 |
| Benzyl Acrylfentanyl | 2,000 | 2,000 | benzyl replacement |  |  | acryl replacement | 2 |
| Acrylfentanyl | 2,000 | 200 |  |  |  | acryl replacement | 1 |
| meta-fluoro Acrylfentanyl | 2,000 | 2,000 |  |  | meta-fluoro substitution | acryl replacement | 2 |
| ortho-fluoro Acrylfentanyl | 2,000 | 2,000 |  |  | ortho-fluoro substitution | acryl replacement | 2 |
| ortho-methyl Acrylfentanyl | 2,000 | 2,000 |  |  | ortho-methyl substitution | acryl replacement | 2 |
| para-chloro Acrylfentanyl | 2,000 | 200 |  |  | para-chloro substitution | acryl replacement | 2 |
| para-fluoro Acrylfentanyl | 2,000 | 2,000 |  |  | para-fluoro substitution | acryl replacement | 2 |
| para-methoxy Acrylfentanyl | 2,000 | ND |  |  | para-methoxy substitution | acryl replacement | 2 |
| para-methyl Acrylfentanyl | 2,000 | 20,000 |  |  | para-methyl substitution | acryl replacement | 2 |
| Benzodioxole fentanyl | 2,000 | ND |  |  |  | benzodioxole substitution | 1 |
| (±)-cis-3-methyl Butyryl fentanyl | 2,000 | 20,000 |  | cis-3-methyl substitution |  | butyryl replacement | 2 |
| Butyryl norfentanyl | 20,000 | 200 | H replacement |  |  | butyryl replacement | 2 |
| α-methyl Butyryl fentanyl | 2,000 | 200 | α-methyl substitution |  |  | butyryl replacement | 2 |
| Butyryl fentanyl | 200 | 200 |  |  |  | butyryl replacement | 1 |
| meta-Fluorobutyryl fentanyl | 2,000 | 2,000 |  |  | meta-fluoro substitution | butyryl replacement | 2 |
| ortho-Fluorobutyryl fentanyl | 2,000 | 2,000 |  |  | ortho-fluoro substitution | butyryl replacement | 2 |
| para-Chlorobutyryl fentanyl | 2,000 | 200 |  |  | para-chloro substitution | butyryl replacement | 2 |
| para-Fluorobutyryl fentanyl | 2,000 | 200 |  |  | para-fluoro substitution | butyryl replacement | 2 |
| para-hydroxy Butyryl fentanyl | 2,000 | 200 |  |  | para-hydroxy substitution | butyryl replacement | 2 |
| para-methyl Butyryl fentanyl | 2,000 | 20,000 |  |  | para-methyl substitution | butyryl replacement | 2 |
| ortho-methoxy Butyryl fentanyl | 2,000 | ND* |  |  | ortho-methoxy substitution | butyryl replacment | 2 |
| para-methoxy Butyryl fentanyl | 2,000 | ND |  |  | para-methoxy substitution | butyryl replacment | 2 |
| Fentanyl Carbamate | 2,000 | 200 |  |  |  | carbamate replacement | 1 |
| Crotonyl fentanyl | 2,000 | 200 |  |  |  | crotonyl replacement | 1 |
| para-fluoro Crotonyl fentanyl | 2,000 | 200 |  |  | para-fluoro substitution | crotonyl replacement | 2 |
| Cyclobutyl fentanyl | 2,000 | 200 |  |  |  | cyclobutyl replacement | 1 |
| para-chloro Cyclobutyl fentanyl | 2,000 | 2,000 |  |  | para-chloro substitution | cyclobutyl replacement | 2 |
| Cyclohexyl fentanyl | 2,000 | ND |  |  |  | cyclohexyl replacement | 1 |
| Cyclopentenyl fentanyl | 2,000 | 20,000 |  |  |  | cyclopentenyl replacement | 1 |
| Cyclopentyl fentanyl | 2,000 | ND |  |  |  | cyclopentyl replacement | 1 |
| para-fluoro Cyclopentyl fentanyl | 2,000 | ND |  |  | para-fluoro substitution | cyclopentyl replacement | 2 |
| para-methyl Cyclopentyl fentanyl | 20,000 | ND |  |  | para-methyl substitution | cyclopentyl replacement | 2 |
| para-chloro Cyclopentyl fentanyl | ND* | ND |  |  | para-chloro substitution | cyclopenyl replacement | 2 |
| Cyclopropaneacetyl fentanyl | 2,000 | 2,000 |  |  |  | cyclopropaneacetyl replacement | 1 |
| N-methyl Cyclopropyl norfentanyl | 2,000 | 200 | H replacement |  |  | cyclopropyl replacement | 2 |
| Cyclopropyl fentanyl | 2,000 | 200 |  |  |  | cyclopropyl replacement | 1 |
| N-benzyl para-fluoro Cyclopropyl norfentanyl | 2,000 | 2,000 | benzyl replacement |  | para-fluoro substitution | cyclopropyl replacement | 3 |
| meta-methyl Cyclopropyl fentanyl | 2,000 | 200 |  |  | meta-methyl substitution | cyclopropyl replacement | 2 |
| ortho-methyl Cyclopropyl fentanyl | 2,000 | 200 |  |  | ortho-methyl substitution | cyclopropyl replacement | 2 |
| para-chloro Cyclopropyl fentanyl | 2,000 | 2,000 |  |  | para-chloro substitution | cyclopropyl replacement | 2 |
| para-fluoro Cyclopropyl fentanyl | 2,000 | 200 |  |  | para-fluoro substitution | cyclopropyl replacement | 2 |
| para-methyl Cyclopropyl fentanyl | 2,000 | 20,000 |  |  | para-methyl substitution | cyclopropyl replacement | 2 |
| Ethoxyacetyl fentanyl | 200 | ND* |  |  |  | ethoxyacetyl replacement | 1 |
| Furanyl fentanyl | 2,000 | 20,000 |  |  |  | furanyl repalcement | 1 |
| N-benzyl Furanyl norfentanyl | 2,000 | 20,000 | benzyl replacement |  |  | furanyl replacement | 2 |
| Furanyl norfentanyl | 20,000 | 2,000 | H replacement |  |  | furanyl replacement | 2 |
| meta-fluoro Furanyl fentanyl | 2,000 | 2,000 |  |  | meta-fluoro substitution | furanyl replacement | 2 |
| meta-methoxy Furanyl fentanyl | 2,000 | ND* |  |  | meta-methoxy substitution | furanyl replacement | 2 |
| meta-methyl Furanyl fentanyl | 2,000 | 20,000 |  |  | meta-methyl substitution | furanyl replacement | 2 |
| ortho-fluoro Furanyl fentanyl | 2,000 | 20,000 |  |  | ortho-fluoro substitution | furanyl replacement | 2 |
| ortho-isopropyl Furanyl fentanyl | 20,000 | ND |  |  | ortho-isopropyl substitution | furanyl replacement | 2 |
| ortho-methoxy Furanyl fentanyl | 2,000 | ND* |  |  | ortho-methoxy substitution | furanyl replacement | 2 |
| ortho-methyl Furanyl fentanyl | 2,000 | 20,000 |  |  | ortho-methyl substitution | furanyl replacement | 2 |
| para-chloro Furanyl fentanyl | 2,000 | 20,000 |  |  | para-chloro substitution | furanyl replacement | 2 |
| para-fluoro Furanyl fentanyl | 2,000 | 2,000 |  |  | para-fluoro substitution | furanyl replacement | 2 |
| para-methoxy Furanyl fentanyl | 2,000 | ND |  |  | para-methoxy substitution | furanyl replacement | 2 |
| para-methyl Furanyl fentanyl | 2,000 | ND* |  |  | para-methyl substitution | furanyl replacement | 2 |
| 4-Anilino-1-Boc-piperidine | ND | ND | tert-butyl acetate replacement |  |  | H replacement | 2 |
| 4-Anilinopiperidine | ND | ND | H replacement |  |  | H replacement | 2 |
| 4-Anilino-1-benzylpiperidine | ND | ND | Benzyl replacement |  |  | H replacement | 2 |
| 4-ANPP | ND | ND |  |  |  | H replacement | 1 |
| Despropionyl 2'-fluoro ortho-Fluorofentanyl | ND | ND | 2'-fluoro substitution |  | ortho-fluoro substitution | H replacement | 3 |
| para-fluoro 4-ANBP | ND | ND | benzyl replacement |  | para-fluoro substitution | H replacement | 3 |
| Despropionyl meta-Methylfentanyl | ND | ND |  |  | meta-methyl substitution | H replacement | 2 |
| Despropionyl ortho-Fluorofentanyl | ND | ND |  |  | ortho-fluoro substitution | H replacement | 2 |
| Despropionyl para-Fluorofentanyl | ND | ND |  |  | para-fluoro substitution | H replacement | 2 |
| Heptanoyl fentanyl | 20,000 | ND |  |  |  | heptanoyl replacment | 1 |
| Hexanoyl fentanyl | 20,000 | ND |  |  |  | hexanoyl replacement | 1 |
| Isobutyryl fentanyl | 2,000 | ND |  |  |  | isobutyryl replacement | 1 |
| FIBF | 2,000 | ND |  |  | para-fluoro substitution | isobutyryl replacement | 2 |
| meta-Fluoroisobutyryl fentanyl | 2,000 | ND |  |  | meta-fluoro substitution | isobutyryl replacement | 2 |
| ortho-Fluoroisobutyryl fentanyl | 2,000 | ND |  |  | ortho-fluoro substitution | isobutyryl replacement | 2 |
| para-Chloroisobutyryl fentanyl | 2,000 | ND* |  |  | para-chloro substitution | isobutyryl replacement | 2 |
| para-methyl Isobutyryl fentanyl | 2,000 | ND |  |  | para-methyl substitution | isobutyryl replacement | 2 |
| Isovaleryl fentanyl | 2,000 | ND |  |  |  | isobvaleryl replacement | 1 |
| Methoxyacetyl fentanyl | 200 | 2,000 |  |  |  | methoxyacetyl replacement | 1 |
| meta-fluoro Methoxyacetyl fentanyl | 200 | 2,000 |  |  | meta-fluoro substitution | methoxyacetyl replacement | 2 |
| meta-methyl Methoxyacetyl fentanyl | 200 | 20,000 |  |  | meta-methyl substitution | methoxyacetyl replacement | 2 |
| Ocfentanil | 200 | 20,000 |  |  | ortho-fluoro substitution | methoxyacetyl replacement | 2 |
| ortho-methyl Methoxyacetyl fentanyl | 200 | 20,000 |  |  | ortho-methyl substitution | methoxyacetyl replacement | 2 |
| para-chloro Methoxyacetyl fentanyl | 2,000 | 2,000 |  |  | para-chloro substitution | methoxyacetyl replacement | 2 |
| para-fluoro Methoxyacetyl fentanyl | 200 | 2,000 |  |  | para-fluoro substitution | methoxyacetyl replacement | 2 |
| para-methoxy Methoxyacetyl fentanyl | 2,000 | ND |  |  | para-methoxy substitution | methoxyacetyl replacement | 2 |
| para-methyl Methoxyacetyl fentanyl | 200 | 20,000 |  |  | para-methyl substitution | methoxyacetyl replacement | 2 |
| Fentanyl Methyl Carbamate | 2,000 | 200 |  |  |  | methyl carbamate replacement | 1 |
| Methacrylfentanyl | 2,000 | 20,000 |  |  |  | methylacryl replacement | 1 |
| N,N-Dimethylamido-despropionyl fentanyl | 200 | 2,000 |  |  |  | N,N-Dimethylamido-despropionyl replacement | 1 |
| para-Toluoyl fentanyl | 20,000 | ND |  |  |  | para-toluoyl replacement | 1 |
| Phenoxyacetyl fentanyl | 2,000 | ND |  |  |  | phenoxyacetyl replacement | 1 |
| N-Benzyl phenyl norfentanyl | 2,000 | ND | benzyl replacement |  |  | phenyl replacement | 2 |
| Phenyl fentanyl | 2,000 | ND |  |  |  | phenyl replacement | 1 |
| ortho-methyl Phenyl fentanyl | 2,000 | ND |  |  | ortho-methyl substitution | phenyl replacement | 2 |
| Phenylacetyl fentanyl | 2,000 | ND |  |  |  | phenylacetyl replacement | 1 |
| Pivaloyl fentanyl | 2,000 | ND |  |  |  | pivaloyl replacement | 1 |
| Senecioylfentanyl | 2,000 | 20,000 |  |  |  | Senecioyl replacement | 1 |
| Tetrahydrofuran fentanyl | 200 | ND |  |  |  | tetrahydrofuran replacement | 1 |
| para-fluoro Tetrahydrofuran fentanyl | 200 | ND |  |  | para-fluoro substitution | tetrahydrofuran replacement | 2 |
| para-methoxy Tetrahydrofuran fentanyl | 2,000 | ND |  |  | para-methoxy substitution | tetrahydrofuran replacement | 2 |
| para-methyl Tetrahydrofuran fentanyl | 2,000 | ND |  |  | para-methyl substitution | tetrahydrofuran replacement | 2 |
| Tetrahydrothiophene fentanyl | 2,000 | ND |  |  |  | tetrahydrothiophene replacement | 1 |
| Thiophene fentanyl | 2,000 | 2,000 |  |  |  | thiophene replacement | 1 |
| Tigloyl fentanyl | 2,000 | ND |  |  |  | tigloyl replacemet | 1 |
| Valeryl fentanyl | 2,000 | 20,000 |  |  |  | valeryl replacement | 1 |
| meta-fluoro Valeryl fentanyl | 2,000 | ND |  |  | meta-fluoro substitution | valeryl replacement | 2 |
| ortho-fluoro Valeryl fentanyl | 2,000 | ND |  |  | ortho-fluoro substitution | valeryl replacement | 2 |
| para-chloro Valeryl fentanyl | 20,000 | ND* |  |  | para-chloro substitution | valeryl replacement | 2 |
| para-fluoro Valeryl fentanyl | 2,000 | ND* |  |  | para-fluoro substitution | valeryl replacement | 2 |
| para-methoxy Valeryl fentanyl | 20,000 | ND |  |  | para-methoxy substitution | valeryl replacement | 2 |
| α'-methoxy Fentanyl | 200 | ND |  |  |  | α'-methoxy substitution | 1 |
| α'-methyl Butyryl fentanyl | 2,000 | ND |  |  |  | α'-methyl butyryl replacement | 1 |
| β'-Phenyl fentanyl | 20,000 | ND |  |  |  | β'-phenyl substitution | 1 |
| (±)-cis-3-methyl Fentanyl | 2,000 | 2,000 |  | (±)-cis-3-methyl substitution |  |  | 1 |
| (±)-trans-3-methyl Fentanyl | 2,000 | 200 |  | (±)-trans-3-methyl substitution |  |  | 1 |
| Norsufentanil | ND | 2,000 | H replacement | 4-methoxymethyl substitution |  |  | 2 |
| Alfentanil | ND | 20,000 | modified tetrazole replacement | 4-methoxymethyl substitution |  |  | 2 |
| Sufentanil | 20,000 | 20,000 | thio replacement | 4-methoxymethyl substitution |  |  | 2 |
| Benzyl Carfentanil | ND | ND | benzyl replacement | 4-methyl acetate substitution |  |  | 2 |
| Norcarfentanil | ND | ND | H replacement | 4-methyl acetate substitution |  |  | 2 |
| Remifentanil | ND | ND | methyl propionate replacement | 4-methyl acetate substitution |  |  | 2 |
| N-methyl Norcarfentanil | ND | ND | methyl replacement | 4-methyl acetate substitution |  |  | 2 |
| Remifentanil Acid | ND | ND | propionic acid | 4-methyl acetate substitution |  |  | 2 |
| Carfentanil | 2,000 | ND |  | 4-methyl acetate substitution |  |  | 1 |
| 4-methyl Fentanyl | 2,000 | 2,000 |  | 4-methyl substitution |  |  | 1 |
| 2,3-seco-Fentanyl | 20,000 | ND |  | broekn ring |  |  | 1 |
| (±)-cis-Isofentanyl | ND | 2,000 | benzyl replacement | cis-3-methyl substitution |  |  | 2 |
| (±)-cis-3-methyl Norfentanyl | ND | 200 | H replacement | cis-3-methyl substitution |  |  | 2 |
| (±)-cis-3-methyl Thiofentanyl | 2,000 | 2,000 | thio replacement | cis-3-methyl substitution |  |  | 2 |
| (±)-trans-3-methyl Thiofentanyl | 2,000 | 200 | thio replacement | trans-3-methyl substitution |  |  | 2 |
| N-(6-APDB) Fentanyl | 2,000 | 200 | 2,3-dihydrobenzofuran replacement; α-methyl substitution |  |  |  | 1 |
| 2',3'-dimethoxy Fentanyl | 2,000 | 200 | 2',3'-dimethoxy substitution |  |  |  | 1 |
| 2',4'-dimethoxy Fentanyl | ND* | 200 | 2',4'-dimethoxy substitution |  |  |  | 1 |
| N-(2,5-DMA) Fentanyl | 2,000 | 200 | 2',5'-dimethoxy subsitution; α-methyl substitution |  |  |  | 1 |
| 2',5'-dimethoxy Fentanyl | 20,000 | 200 | 2',5'-dimethoxy substitutio |  |  |  | 1 |
| N-(2C-G) Fentanyl | ND | 200 | 2',5'-dimethoxy; 3',4'-dimethyl substitution |  |  |  | 1 |
| N-(2C-B) Fentanyl | 20,000 | 2,000 | 2',5'-dimethoxy; 4'-bromo substitution |  |  |  | 1 |
| N-(DOB) Fentanyl | ND | 2,000 | 2',5'-dimethoxy; 4'-bromo substitution; α-methyl substitution |  |  |  | 1 |
| N-(DOBU) Fentanyl | ND | 2,000 | 2',5'-dimethoxy; 4'-butyryl substitution; α-methyl substitution |  |  |  | 1 |
| N-(2C-C) Fentanyl | 20,000 | 2,000 | 2',5'-dimethoxy; 4'-chloro substitution |  |  |  | 1 |
| N-(2C-E) Fentanyl | ND | 2,000 | 2',5'-dimethoxy; 4'-ethyl substitution |  |  |  | 1 |
| N-(DOET) Fentanyl | ND | 2,000 | 2',5'-dimethoxy; 4'-ethyl substitution; α-methyl substitution |  |  |  | 1 |
| N-(2C-T-2) Fentanyl | ND | 200 | 2',5'-dimethoxy; 4'-ethyl sulfide substitution |  |  |  | 1 |
| N-(DOI) Fentanyl | ND | 2,000 | 2',5'-dimethoxy; 4'-iodo substitution; α-methyl substitution |  |  |  | 1 |
| N-(2C-iP) Fentanyl | ND | 2,000 | 2',5'-dimethoxy; 4'-isopropyl substitution |  |  |  | 1 |
| N-(2C-T-4) Fentanyl | ND | 2,000 | 2',5'-dimethoxy; 4'-isopropyl sulfide substitution |  |  |  | 1 |
| N-(2C-D) Fentanyl | ND | 200 | 2',5'-dimethoxy; 4'-methyl substitution |  |  |  | 1 |
| N-(2C-T) Fentanyl | ND | 200 | 2',5'-dimethoxy; 4'-methyl sulfide substitution |  |  |  | 1 |
| N-(2C-N) Fentanyl | ND | 200 | 2',5'-dimethoxy; 4'-nitro substitution |  |  |  | 1 |
| N-(2C-T-7) Fentanyl | ND | 2,000 | 2',5'-dimethoxy; 4'-propyl sulfide substitution |  |  |  | 1 |
| N-(2C-TFM) Fentanyl | ND | 2,000 | 2',5'-dimethoxy; 4'-trifluoromethyl substitution |  |  |  | 1 |
| 2',6'-dimethoxy Fentanyl | 20,000 | 200 | 2',6'-dimethoxy substitutio |  |  |  | 1 |
| 2'-Fluorofentanyl | 2,000 | 200 | 2'-fluoro substitution |  |  |  | 1 |
| 2'-methyl Fentanyl | 2,000 | 200 | 2'-methyl substitution |  |  |  | 1 |
| N-(3,4,5-TMA) Fentanyl | ND | 200 | 3',4',5'-trimethoxy substitution; α-methyl substitution |  |  |  | 1 |
| 3',4'-dimethoxy Fentanyl | ND | 200 | 3',4'-dimethoxy substitution |  |  |  | 1 |
| 3',5'-dimethoxy Fentanyl | ND | 2,000 | 3',5'-dimethoxy substitution |  |  |  | 1 |
| N-(DOC) Fentanyl | ND | 200 | 3',5'-dimethoxy; 4'-chloro substitution; α-methyl substitution |  |  |  | 1 |
| N-(2C-I) Fentanyl | ND* | 2,000 | 3',5'-dimethoxy; 4'-iodo substitution |  |  |  | 1 |
| N-(DOM) Fentanyl | ND | 200 | 3',5'-dimethoxy; 4'-methyl substitution; α-methyl substitution |  |  |  | 1 |
| N-(2C-P) Fentanyl | ND | 20,000 | 3',5'-dimethoxy; 4'-propyl substitution |  |  |  | 1 |
| N-(3-ethylindole) Norfentanyl | 2,000 | 200 | 3-ethylindole replacement |  |  |  | 1 |
| 3'-Fluorofentanyl | 2,000 | 200 | 3'-fluoro substitution |  |  |  | 1 |
| 3'-methyl Fentanyl | 2,000 | 200 | 3'-methyl substitution |  |  |  | 1 |
| N-(2C-B-fly) Fentanyl | ND | 2,000 | 4-bromo-2,3,6,7-tetrahydrobenzo[1,2-b:4,5-b']difuran replacement |  |  |  | 1 |
| N-(3C-B-fly) Fentanyl | ND | 200 | 4-bromo-2,3,6,7-tetrahydrobenzo[1,2-b:4,5-b']difuran replacement; α-methyl substitution |  |  |  | 1 |
| 4'-Fluorofentanyl | 2,000 | 200 | 4'-fluoro substitution |  |  |  | 1 |
| 4'-methyl Fentanyl | 2,000 | 200 | 4'-methyl substitution |  |  |  | 1 |
| N-(MDA) Fentanyl | 2,000 | 200 | benzodioxole replacement; α-methyl substitution |  |  |  | 1 |
| N-(2-APB) Fentanyl | 20,000 | 200 | benzofuran replacement; α-methyl substitution |  |  |  | 1 |
| N-(6-APB) Fentanyl | 2,000 | 200 | benzofuran replacement; α-methyl substitution |  |  |  | 1 |
| Benzyl fentanyl | 2,000 | 200 | benzyl replacement |  |  |  | 1 |
| Furanylethyl fentanyl | 2,000 | 200 | furanylethyl replacement |  |  |  | 1 |
| Norfentanyl | 20,000 | 200 | H replacement |  |  |  | 1 |
| N-methyl Norfentanyl | 2,000 | 200 | methyl replacement |  |  |  | 1 |
| Thienyl fentanyl | 2,000 | 200 | thienyl replacement |  |  |  | 1 |
| Thiofentanyl | 2,000 | 200 | thio replacement |  |  |  | 1 |
| α-methyl Thiofentanyl | 2,000 | 200 | thio replacement; α-methyl substitution |  |  |  | 1 |
| β-Hydroxythiofentanyl | 200 | 200 | thio replacement; β-hydroxy substitution |  |  |  | 1 |
| N-(Phentermine) Fentanyl | 2,000 | 200 | α-dimethyl substitution |  |  |  | 1 |
| α-methyl Fentanyl | 2,000 | 200 | α-methyl substitution |  |  |  | 1 |
| β-hydroxy Fentanyl | 200 | 200 | β-hydroxy substitution |  |  |  | 1 |
| β-methyl Fentanyl | 2,000 | 200 | β-methyl substitution |  |  |  | 1 |
| Fentanyl | 200 | 200 |  |  |  |  | 0 |
| 4'-fluoro, para-fluoro (±)-trans-3-methyl Fentanyl | 2,000 | 2,000 | 4'-fluoro substitution | trans-3-methyl substitution | para-fluoro substitution |  | 3 |
| 2'-fluoro ortho-Fluorofentanyl | 2,000 | 2,000 | 2'-fluoro substitution |  | ortho-fluoro substitution |  | 2 |
| 3'-fluoro ortho-Fluorofentanyl | 2,000 | 200 | 3'-fluoro substitution |  | ortho-fluoro substitution |  | 2 |
| N-benzyl para-fluoro Norfentanyl | 2,000 | 200 | benzyl replacement |  | para-fluoro substitution |  | 2 |
| 4-Phenyl fentanyl | ND* | ND |  |  | 4-phenyl substitution |  | 1 |
| meta-Fluorofentanyl | 2,000 | 200 |  |  | meta-fluoro substitution |  | 1 |
| meta-Methylfentanyl | 2,000 | 2,000 |  |  | meta-methyl substitution |  | 1 |
| ortho-Fluorofentanyl | 2,000 | 2,000 |  |  | ortho-fluoro substitution |  | 1 |
| ortho-Methylfentanyl | 2,000 | 200 |  |  | ortho-methyl substitution |  | 1 |
| para-Bromofentanyl | 2,000 | 200 |  |  | para-bromo substitution |  | 1 |
| para-Chlorofentanyl | 2,000 | 200 |  |  | para-chloro substitution |  | 1 |
| para-Fluorofentanyl | 200 | 200 |  |  | para-fluoro substitution |  | 1 |
| para-Methoxyfentanyl | 2,000 | ND* |  |  | para-methoxy substitution |  | 1 |
| para-Methylfentanyl | 2,000 | 2,000 |  |  | para-methyl substitution |  | 1 |

# **Table S3**: modifications that cause non-detection for both BTNX and DanceSafe FTS

| **Piperidine modifications** | | | |
| --- | --- | --- | --- |
| Structure | Modification | # compounds with mod | Compounds with modification |
|  | 4-phenyl substitution | 1 | **4-Phenyl fentanyl** |
|  |  |  |  |
|  | H replacement | 7 | **4-ANPP**, Despropionyl meta-Methylfentanyl, Despropionyl ortho-Fluorofentanyl, Despropionyl para-Fluorofentanyl, 4-Anilino-1-Boc-piperidine, Despropionyl 2'-fluoro ortho-Fluorofentanyl, para-fluoro 4-ANBP |

# **Table S4**: modifications that cause non-detection for DanceSafe FTS without any co-occurring modifications

| **Piperidine modifications** | | | | |
| --- | --- | --- | --- | --- |
| Structure | modification | # with mod | Compounds with modification |  |
|  | 4-methyl acetate substitution | 6 | **Carfentanil,** Benzyl Carfentanil, Remifentanil, Norcarfentanil,  N-methyl Norcarfentanil,  Remifentanil Acid |  |
|  | Broken ring | 1 | **2,3-seco-Fentanyl** |  |
| **Aniline modifications** | | | | |
|  | Para-methoxy substitution | 8 | **para-Methoxyfentanyl**  para-methoxy Acetyl fentanyl  para-methoxy Acrylfentanyl  para-methoxy Butyryl fentanyl  para-methoxy Furanyl fentanyl  para-methoxy Methoxyacetyl fentanyl  para-methoxy Tetrahydrofuran fentanyl  para-methoxy Valeryl fentanyl |  |
| **Carbonyl modifications** | | | | |
|  | Isobutyryl replacement | 6 | **Isobutyryl fentanyl**  meta-Fluoroisobutyryl fentanyl  ortho-Fluoroisobutyryl fentanyl  para-Chloroisobutyryl fentanyl  FIBF  para-methyl Isobutyryl fentanyl |  |
|  | Tetrahydrofuran replacement | 4 | **Tetrahydrofuran fentanyl**  para-fluoro Tetrahydrofuran fentanyl  para-methoxy Tetrahydrofuran fentanyl  para-methyl Tetrahydrofuran fentanyl |  |
|  | Cyclopentyl replacement | 4 | **Cyclopentyl fentanyl**  para-fluoro Cyclopentyl fentanyl  para-methyl Cyclopentyl fentanyl  para-chloro Cyclopentyl fentanyl |  |
|  | Phenyl replacement | 2 | **Phenyl fentanyl**  ortho-methyl Phenyl fentanyl |  |
|  | β’-phenyl substitution | 1 | **β'-Phenyl fentanyl** |  |
|  | α’-methyl butyryl replacement | 1 | **α'-methyl Butyryl fentanyl** |  |
|  | α’-methoxy substitution | 1 | **α'-methoxy Fentanyl** |  |
|  | tigloyl replacement | 1 | **Tigloyl fentanyl** |  |
|  | tetrahydrothiophene replacement | 1 | Tetrahydrothiophene fentanyl |  |
|  | Pivaloyl replacement | 1 | **Pivaloyl fentanyl** |  |
|  | Phenylacetyl replacement | 1 | **Phenylacetyl fentanyl** |  |
|  | phenoxyacetyl replacement | 1 | **Phenoxyacetyl fentanyl** |  |
|  | para-toluoyl replacement | 1 | para-Toluoyl fentanyl |  |
|  | isovaleryl replacement | 1 | **Isovaleryl fentanyl** |  |
|  | Hexanoyl replacement | 1 | **Hexanoyl fentanyl** |  |
|  | Heptanoyl replacement | 1 | **Heptanoyl fentanyl** |  |
|  | ethoxyacetyl replacement | 1 | **Ethoxyacetyl fentanyl** |  |
|  | cyclohexyl replacement | 1 | **Cyclohexyl fentanyl** |  |
|  | Benzodioxole replacement | 1 | Benzodioxole fentanyl |  |
|  | 3-thiophene replacement | 1 | **Thiophene fentanyl 3-thiophenecarboxamide** |  |
|  | 2,2,3,3-tetramethyl cyclopropyl replacement | 1 | 2,2,3,3-tetramethyl-Cyclopropyl fentanyl |  |
|  | 2,3-benzodioxole substitution | 1 | 2,3-Benzodioxole fentanyl |  |

# **Figure S3**: UpSet plot of all compounds that are not detectable by DanceSafe FTS


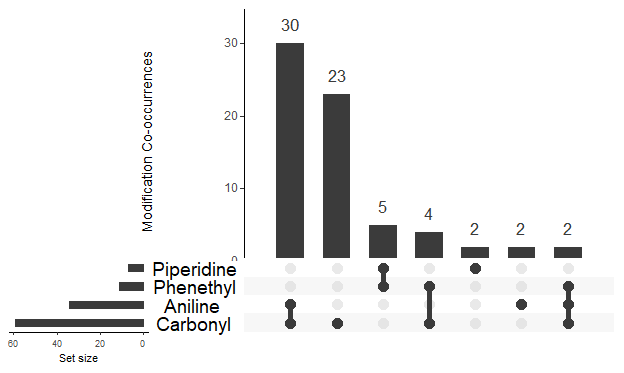


# **Figure S4**: Carbonyl modifications that do NOT cause non-detects for DanceSafe (on their own, without any co-occurring modifications)


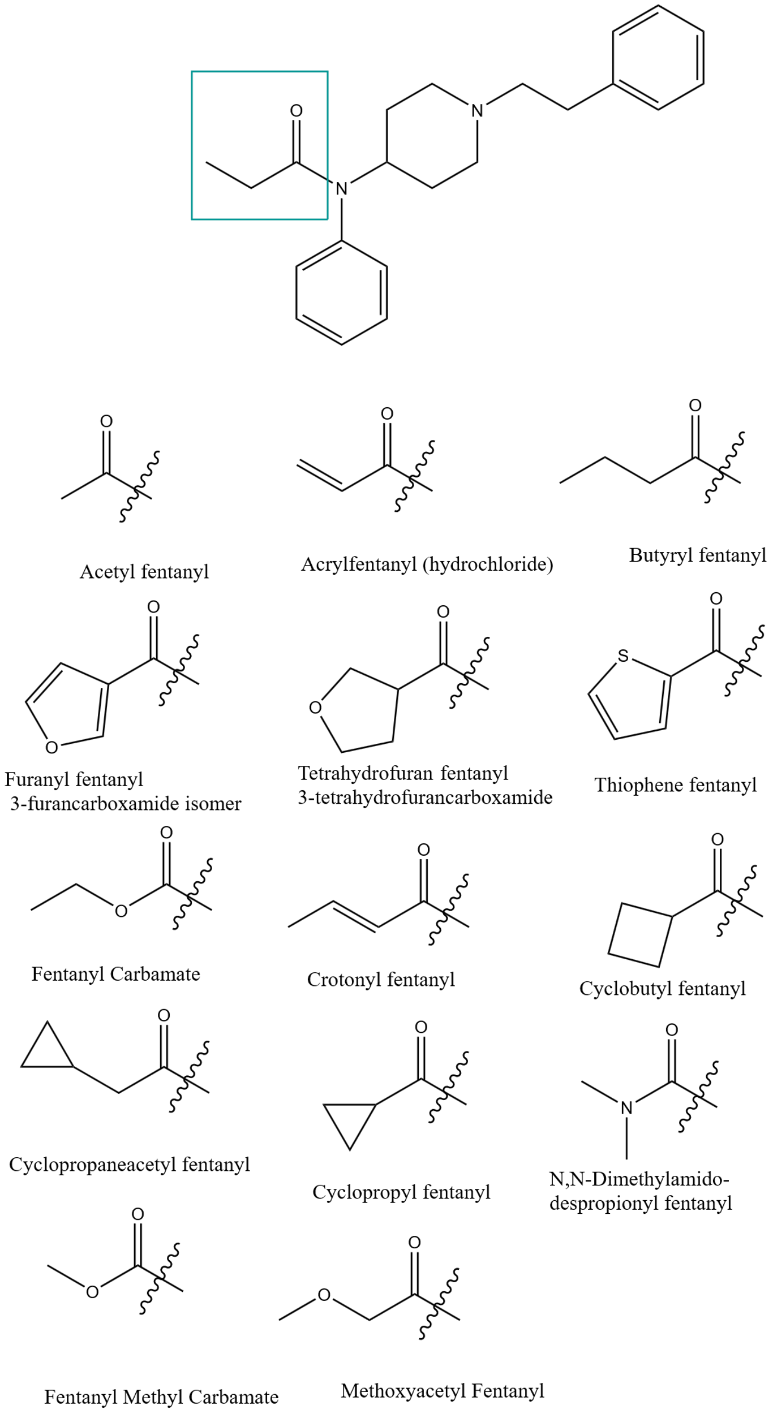


# **Figure S5**: UpSet plot of co-modifications that cause non-detection for BTNX FTS (including compounds ND by DanceSafe as well)


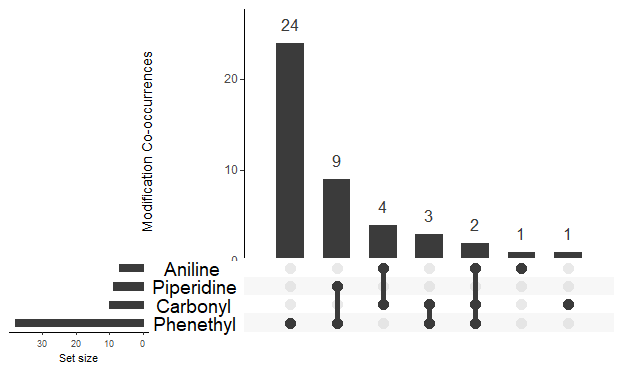


# **Figure S6**: Phenethyl modifications that **inhibit** BTNX detection


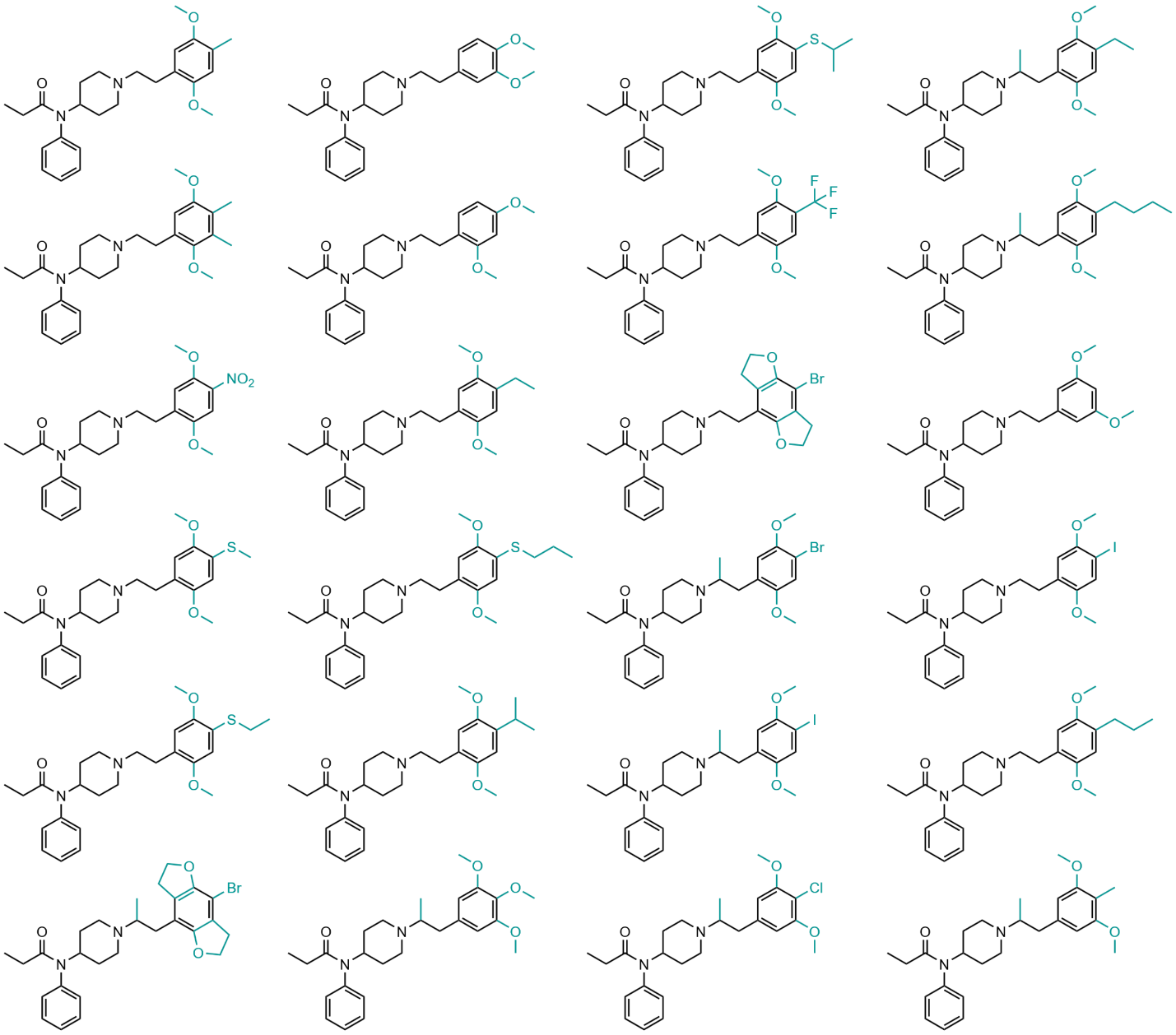


# **Figure S7**: Phenethyl modifications that **do not** inhibit BTNX detection


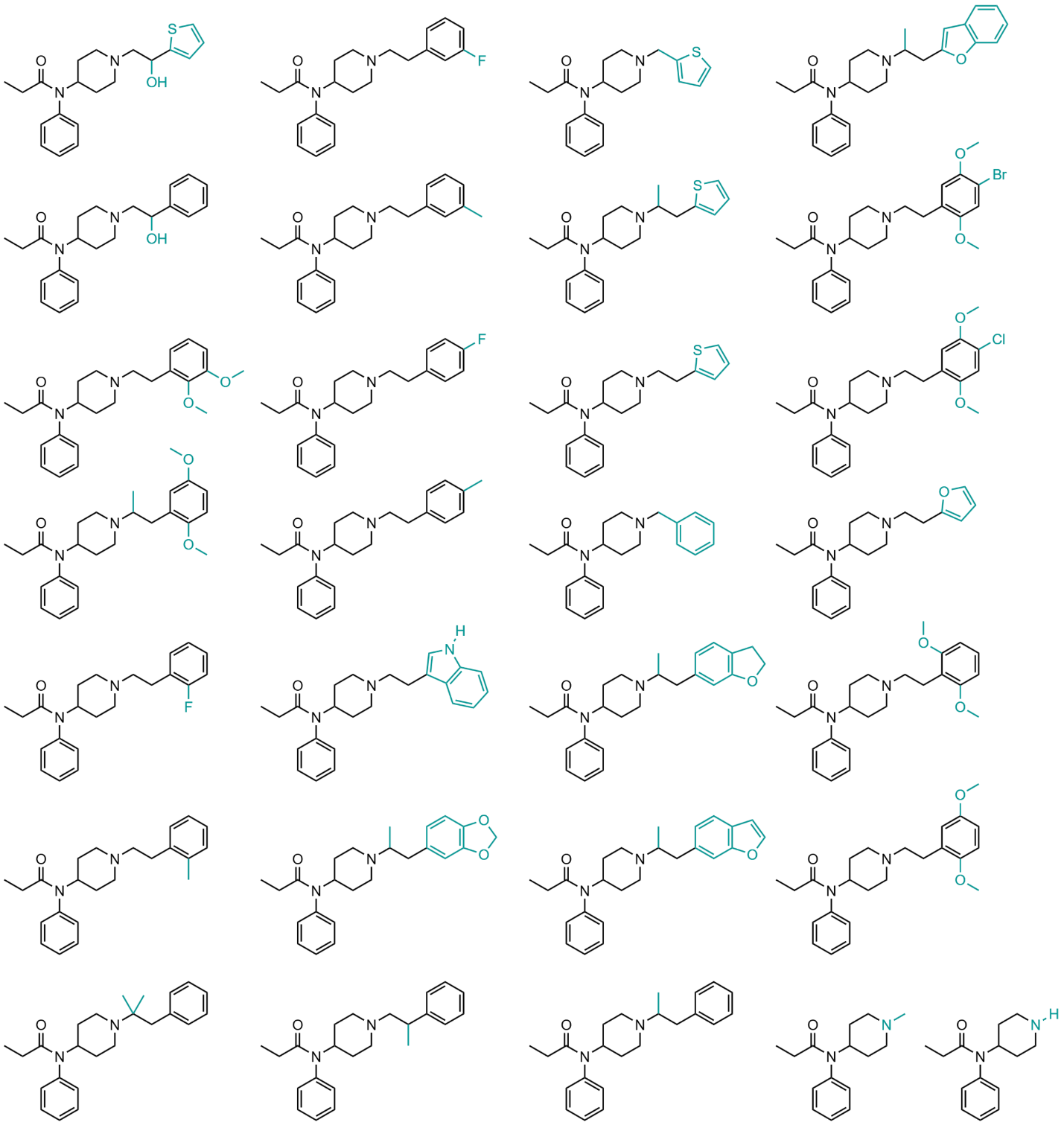


# **Figure S8**: left: N-(2C-B) Fentanyl (top) and N-(2C-C) Fentanyl (bottom) are detectable by BTNX FTS; right: structurally similar compounds N-(DOB) fentanyl (top) and N-(DOC) fentanyl (bottom) and N-(2C-I) Fentanyl (right) are not detectable by BTNX FTS


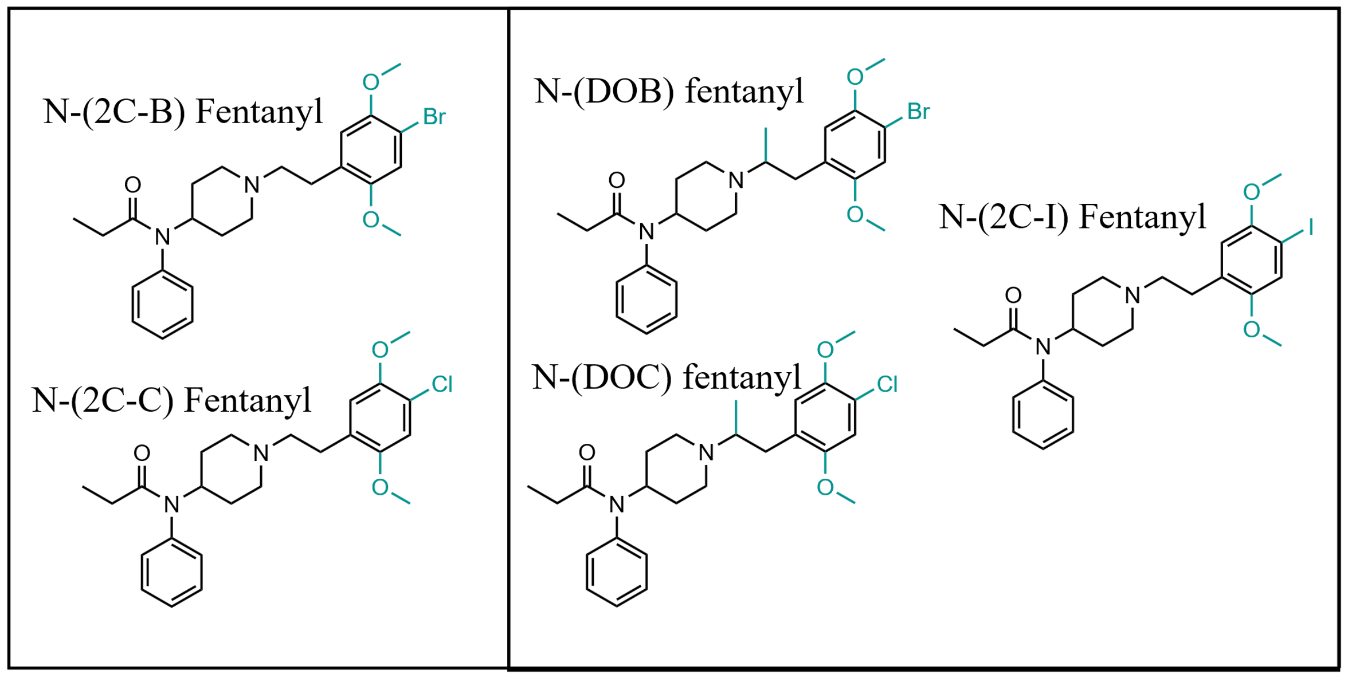

Supplement: Supplementary file 1 — Additional file 1: This file contains supporting information, including the limits of detection, structures, and modifications of all compounds examined in this study and additional UpSet plots illustrating co-modifications. [file 12954_2023_911_MOESM1_ESM.docx]
